# The immune microenvironment in EGFR- and ERBB2-mutated lung adenocarcinoma

**DOI:** 10.1016/j.esmoop.2021.100253

**Published:** 2021-09-03

**Authors:** M. Kirchner, K. Kluck, R. Brandt, A.-L. Volckmar, R. Penzel, D. Kazdal, V. Endris, O. Neumann, H. Seker-Cin, H. Goldschmid, J. Glade, M. Allgäuer, M. Kriegsmann, H. Winter, T. Muley, S. Perner, N. Frost, M. Reck, S. Fröhling, P. Schirmacher, M. Thomas, J. Budczies, P. Christopoulos, A. Stenzinger

**Affiliations:** 1 Institute of Pathology, Heidelberg University Hospital, Heidelberg, Germany; 2 German Cancer Consortium (DKTK) and German Cancer Research Center (DKFZ), Heidelberg, Germany; 3 Translational Lung Research Center Heidelberg (TLRC-H), Heidelberg, Germany; 4 Department of Thoracic Surgery, Thoraxklinik at Heidelberg University Hospital, Heidelberg, Germany; 5 Translational Research Unit, Thoraxklinik at Heidelberg University Hospital, Heidelberg, Germany; 6 Pathology of the University Medical Center Schleswig-Holstein (UKSH), Campus Lübeck and the Research Center Borstel, Borstel, Germany; 7 Airway Research Center North (ARCN), Borstel, Germany; 8 Department of Infectious Diseases and Respiratory Medicine, Charité-Universitätsmedizin Berlin, Berlin, Germany; 9 Berlin Institute of Health, Berlin, Germany; 10 Department of Thoracic Oncology, Lung Clinic Grosshansdorf, Grosshansdorf, Germany; 11 Department of Translational Medical Oncology, National Center for Tumor Diseases (NCT), Heidelberg, Germany; 12 Thoraxklinik and National Center for Tumor Diseases (NCT) at Heidelberg University Hospital, Heidelberg, Germany

**Keywords:** lung adenocarcinoma, EGFR exon 20 insertion, ERBB2 exon 20 insertion, immunosuppression, tumor microenvironment

## Abstract

**Background:**

Targeted therapies have improved survival and quality of life for patients with non-small-cell lung cancer with actionable driver mutations. However, epidermal growth factor receptor (*EGFR*) and human epidermal growth factor receptor 2 gene (*HER2*, also known as *ERBB2*) exon 20 insertions (Ex20mut) are characterized by a poor response to currently approved tyrosine kinase inhibitors and immunotherapies. The underlying immune biology is not well understood.

**Materials and methods:**

We carried out messenger RNA expression profiling of lung adenocarcinomas (ADCs) with *ERBB2* (*n* = 19) and *EGFR* exon 20-insertion mutations (*n* = 13) and compared these to tumors with classical *EGFR* mutations (*n* = 40, affecting *EGFR* exons 18, 19 or 21) and *EGFR/ERBB2* mutation-negative lung ADC (EGFR/ERBB2wt, *n* = 26) focusing on immunologically relevant transcripts. Tumor-infiltrating immune cells were estimated from gene expression profiles.

**Results:**

Cytotoxic cells were significantly lower in *EGFR*-mutated tumors regardless of the affected exon, while Th1 cells were significantly lower in EGFR-Ex20mut compared to EGFR/ERBB2wt tumors. We assessed the differentially expressed genes of ERBB2-Ex20mut and EGFR-Ex20mut tumors compared to EGFR-Ex18/19/21mut and EGFR/ERBB2wt tumors. Of these, the genes *GUSB*, *HDAC11*, *IFNGR2*, *PUM1*, *RASGRF1* and *RBL2* were up-regulated, while a lower expression of *CBLC*, *GBP1*, *GBP2*, *GBP4* and *MYC* was observed in all three comparison groups. The omnibus test revealed 185 significantly (FDR = 5%) differentially expressed genes and we found these four most significant gene expression changes in the study cohort: *VHL* and *JAK1* were overexpressed in ERBB2-Ex20mut and EGFR-Ex20mut tumors compared to both EGFR-Ex18/19/21mut and EGFR/ERBB2wt tumors. *RIPK1* and *STK11IP* showed the highest expression in ERBB2-Ex20mut tumors.

**Conclusions:**

Targeted gene expression profiling is a promising tool to read out the characteristics of the tumor microenvironment from routine diagnostic lung cancer biopsies. Significant immune reactivity and specific immunosuppressive characteristics in ERBB2-Ex20mut and EGFR-Ex20mut lung ADC with at least some degree of immune infiltration support further clinical evaluation of immune-modulators as partners of immune checkpoint inhibitors in such tumors.

## Highlights

• Gene expression profiling to characterize the tumor microenvironment is feasible using diagnostic lung cancer biopsies.
• EGFR exon 20-mutated tumors show a higher expression of *VHL* and an immunologic ‘colder’ phenotype than EGFR/ERBB2wt tumors.
• ERBB2 exon 20-mutated tumors show an overexpression of *RIPK1* and *STK11IP* and a reduction of cytotoxic natural killer cells.
• Drugs targeting these alterations are potential partners of checkpoint blockade in exon 20-mutated non-small-cell lung cancer.

## Introduction

Non-small-cell lung cancer (NSCLC) is the leading cause of cancer-related mortality worldwide.^1^ By means of genetic profiling, the molecular classification of advanced NSCLC based on oncogenic driver mutations became feasible. Targeted therapies, mainly tyrosine kinase inhibition (TKI), have improved the overall survival and quality of life for patients with actionable driver mutations.^2^ Epidermal growth factor receptor (*EGFR*)-activating mutations represent the most frequent targetable alteration with a prevalence of nearly 20% in Caucasians with lung adenocarcinomas (ADCs) and show sensitivity to various TKIs.^3^ However, exon 20 insertions, which account for 1%-10% of all *EGFR* mutations, define a distinct subset of lung ADC characterized by a poor response to all currently approved EGFR-TKIs.^4^^,^^5^ Similarly, 12-bp in-frame insertions and other mutations of the human epidermal growth factor receptor 2 gene (*HER2*, also known as *ERBB2*) are oncogenic drivers in 2%-3% of NSCLC^6^, ^7^, ^8^ that are resistant to EGFR-TKIs and difficult to target with specific ERBB2 and dual EGFR/ERBB2 inhibitors, as they result in steric hindrance of the drug-binding pocket.^9^^,^^10^

Another potential therapeutic approach is the use of immune checkpoint inhibitors (ICIs).^11^, ^12^, ^13^ However, the efficacy of immunotherapy in driver-dependent NSCLC is inferior, possibly due to oncogene-induced alterations of the tumor microenvironment (TME).^14^ The biological basis for this partial response to ICI is poorly understood, but it is interesting that ICI sensitivity appears to vary by type of *EGFR* mutation in NSCLC. Some tumors with uncommon *EGFR* mutations, including exon 20 insertions, show better responses than tumors with common *EGFR* mutations.^15^ TME composition is generally recognized as a crucial parameter for the efficacy of ICIs, but biological data, especially for the patients with NSCLC with uncommon *EGFR* or *ERBB2* mutations, are scarce.^16^^,^^17^

We employed the NanoString nCounter technology (NanoString Technologies, Seattle, WA) with the PanCancer Human IO 360 Panel to investigate the TME in 98 formalin-fixed and paraffin-embedded (FFPE) biopsies of clinically annotated ERBB2 exon 20-positive, EGFR exon 20-positive, EGFR exon 18/19/21-positive and EGFR/ERBB2-negative advanced lung ADC.

## Materials and methods

### Study cohort

This retrospective study cohort included all ERBB2 exon 20-positive and EGFR exon 20-positive tumors with available material and appropriate RNA quality among patients diagnosed and treated at the Heidelberg University Hospital between 2007 and 2020 (Table 1). In addition, 40 EGFR exon 18/19/21-positive and 26 EGFR/ERBB2-negative (EGFR/ERBB2wt) lung ADC were analyzed as controls. ERBB2 and EGFR status was determined at the Heidelberg Institute of Pathology using our routine diagnostic workflow of combined DNA and RNA sequencing starting from FFPE lung biopsies.^18^ Tumors harboring *ERBB2* exon 20 insertions were classified as *ERBB2* exon 20 positive (ERRB2-Ex20mut), tumors harboring *EGFR* exon 20 insertions were classified as *EGFR* exon 20 positive (EGFR-Ex20mut) and tumors harboring activating mutations in exon 18, 19 or 21 of *EGFR* (EGFR-Ex18/19/21mut) were classified as *EGFR* exon 18/19/21 positive (Supplementary Table S1, available at https://doi.org/10.1016/j.esmoop.2021.100253).

**Table 1:** Clinicopathological characteristics of the study cohort comprising 98 lung adenocarcinomas

Variable	ERBB2-Ex20mut	EGFR-Ex20mut	EGFR-Ex18/19/21mut	EGFR/ERBB2wt
Total number	19	13	40	26
Age, years, median (min-max)	69 (40-84)	71 (52-83)	69.5 (46-83)	65.5 (53-89)
Sex, *n* (%)				
Male	4 (21)	4 (31)	7 (17.5)	13 (50)
Female	15 (79)	9 (69)	33 (82.5)	13 (50)
Stage, *n* (%)				
I	3 (16)	0 (0)	0 (0)	0 (0)
II	1 (5)	1 (8)	4 (10)	0 (0)
III	4 (21)	1 (8)	10 (25)	0 (0)
IV	11 (58)	11 (84)	26 (65)	26 (100)
Prior therapy, *n* (%)				
Naïve	19 (100)	13 (100)	40 (100)	26 (100)
Chemotherapy	0 (0)	0 (0)	0 (0)	0 (0)

All patients in this cohort were therapy-naïve, i.e. received neither TKI nor chemo- or immunotherapy before biopsy. For all patients, only biopsies from the primary (lung) tumor with sufficient available messenger RNA (mRNA) for expression profiling were analyzed. The study was approved by the ethics committee of Heidelberg University (S-145/2017). Part of the sub-cohort of EGFR/ERBB2wt tumors was also analyzed in two earlier studies characterizing the TME in different patients with lung ADC.^19^^,^^20^

### Targeted gene expression profiling

RNA extracts passing the following steps of quality control were considered as suitable for gene expression analysis: RNA concentration of at least 10 ng/μl, sufficient RNA purity with an A260/A280 in the range of 1.7-2.3 and sufficient RNA integrity with at least 90% of the fragments longer than 100 nucleotides. Targeted mRNA expression profiling was conducted on the NanoString nCounter gene expression platform (NanoString Technologies) using the PanCancer Human IO 360 Panel as described before.^19^^,^^20^

### Data processing

Statistical analysis and graphics generation were carried out using the programming language R (R Foundation for Statistical Computing, Vienna, Austria). Analysis of expression data, estimation of the abundance of 14 immune cell populations [B cells, CD45+ cells, CD56dim natural killer (NK) cells, CD8+ T cells, cytotoxic cells, dendritic cells, exhausted CD8+ T cells, macrophages, mast cells, neutrophils, NK cells, T cells, Th1 cells and regulatory T (Treg) cells],^20^^,^^21^ calculation of the total score of tumor-infiltrating lymphocytes (total TILs), hierarchical clustering and heatmap displays were carried out as described before.^19^^,^^20^ Abundances (or expression levels) above the mean appear in yellow, and abundances below the mean in blue. Correlations between clusters and genetic subgroups were assessed using Fisher’s exact test.

A gene set enrichment analysis was carried out by annotation categories given by NanoString. Significant enrichments or depletions of groups of genes were assessed using Fisher’s exact test.

Differences between ERRB2-Ex20mut, EGFR-Ex20mut, EGFR-Ex18/19/21mut and EGFR/ERBB2wt tumors were assessed for significance using the Kruskal–Wallis test as omnibus test and the Wilcoxon test as post hoc test. The Benjamini–Hochberg procedure was used for *P* value correction, and lists of cell populations or genes were compiled controlling the false discovery rate (FDR) at 5%. KEGG Mapper was used to visualize the signaling pathways in cancer and the cytokine–cytokine receptor network (pathway hsa05200 and hsa04060).^22^

## Results

Overall, 19 ERRB2-Ex20mut and 13 EGFR-Ex20mut tumors could be included in the study and compared to 40 EGFR-Ex18/19/21mut and 26 EGFR/ERBB2wt lung ADC samples (Table 1). Biopsies of each of the 98 primary tumors underwent gene expression profiling with an assay of 770 genes focused on immune-related genes. The abundance of 14 immune cell populations in the TME was estimated from the mRNA expression of marker genes and was reported on log2 scale as described before.^20^^,^^21^

### Overall level of immune cell infiltration

**Figure 1:**
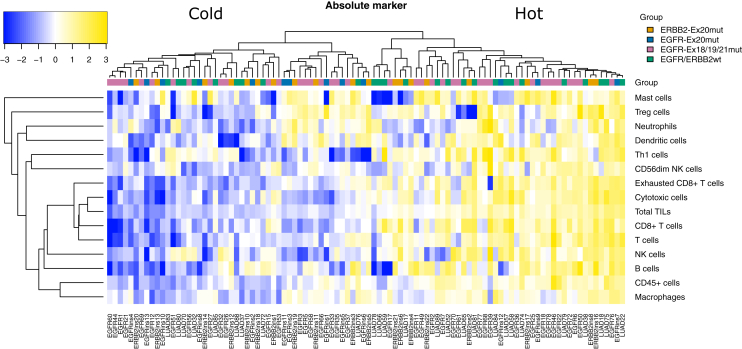
Immunological analysis of 98 lung adenocarcinomas by targeted gene expression profiling. Clustering of the tumors by the abundance of 14 immune cell populations. NK, natural killer; TILs, tumor-infiltrating lymphocytes; Treg, regulatory T.

The levels of the immune cell populations were grouped by hierarchical clustering and visualized in a heatmap (Figure 1). The markers of cytotoxic cells, total TILs, T cells, CD8+ T cells and exhausted CD8+ T cells clustered tightly together (all pairwise Spearman correlations ρ > 0.74 with *P* < 2.2 × 10^−16^). Moreover, macrophages and CD45+ cells showed a strong positive correlation (ρ = 0.66, *P* < 2.2 × 10^−16^). The tumors clustered together in two main immunological groups, ‘cold’ tumors (*n* = 56) and ‘hot’ tumors (*n* = 42). The ‘cold’ phenotype did not correlate with the mutational status of the tumors (*P* = 0.233), but was the predominant pattern in 60%-77% of cases across the mutated sample groups: 68% (13/19) of the ERRB2-Ex20mut samples, 77% (10/13) of the EGFR-Ex20mut samples and 60% (24/40) of the EGFR-Ex18/19/21mut samples could be assigned to the category of immunologically ‘cold’ tumors. In contrast, 32% (6/19), 23% (3/13) and 40% (16/40), respectively, could be assigned to the immunologically ‘hot’ tumors. At the same time, there was a clear trend for all mutated tumor samples collectively to show a colder phenotype compared to EGFR/ERBB2wt group (*P* = 0.068), the majority of which could be assigned to the immunological group of ‘hot’ tumors (17/26 = 65% versus 9/26 = 35%). While no significant difference could be shown regarding the immunological categories between ERRB2-Ex20mut samples (*P* = 0.141), EGFR-Ex18/19/21mut samples (*P* = 0.310) and EGFR/ERBB2wt samples, there was a significant difference between EGFR-Ex20mut samples and EGFR/ERBB2wt samples (*P* = 0.018).

Of note, the levels of CD45+ cells and total TILs (calculated as in Danaher et al.^21^) did not correlate with the mutation type, either (*P* = 0.46 and *P* = 0.10).

### Specific immune cell populations

**Figure 2:**
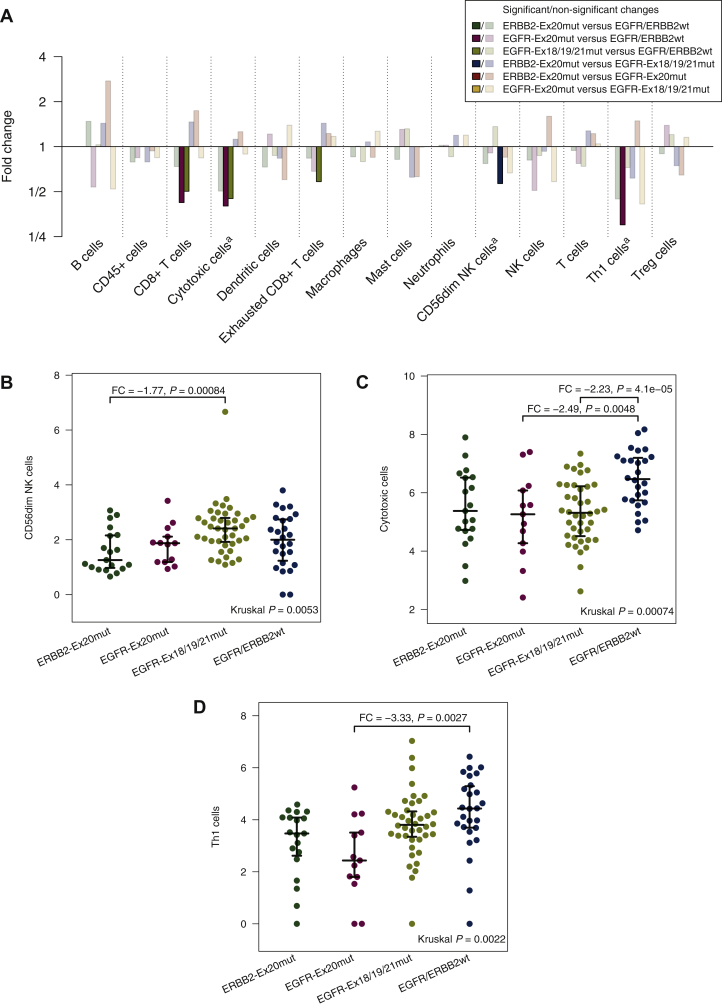
Fold changes (FCs), respectively, absolute levels of specific immune cell populations. (A) FCs of the immune cell levels between ERBB2-Ex20mut, EGFR-Ex20mut, EGFR-Ex18/19/21mut and EGFR/ERBB2wt lung adenocarcinomas. Brightly colored bars show significant differences. ^a^Significant in omnibus test. (B-D) Absolute levels of CD56dim NK cells, cytotoxic cells and Th1 cells. Distributions are shown with median, lower and upper quartile. If there are significant differences, the FC and the *P* value are given above the respective bracket. (B) Significantly lower CD56dim NK cells in ERBB2-Ex20mut compared to EGFR-Ex18/19/21mut tumors. (C) Significantly lower cytotoxic cells in EGFR-Ex20mut and EGFR-Ex18/19/21mut compared to EGFR/ERBB2wt tumors. (D) Significantly lower Th1 cells in EGFR-Ex20mut compared to EGFR/ERBB2wt tumors. NK, natural killer; Treg, regulatory T.

Three of 14 immune cell populations were significantly different in ERRB2-Ex20mut, EGFR-Ex20mut, EGFR-Ex18/19/21mut and EGFR/ERBB2wt tumors using omnibus testing and multiple testing correction (Figure 2A, marked by ^a^). CD56dim NK cells were significantly lower in ERRB2-Ex20mut compared to EGFR-Ex18/19/21mut tumors [Figure 2B; fold change (FC) = −1.8, *P* = 0.00084]. Cytotoxic cells were significantly lower in EGFR-Ex20mut and EGFR-Ex18/19/21mut compared to EGFR/ERBB2wt tumors (Figure 2C; FC = −2.5, *P* = 0.0048 and FC = −2.2, *P* = 4.1E−05). Here, ‘cytotoxic cells’ (marker genes: *PRF1*, *GZMA*, *GZMB*, *GZMH*, *GNLY*, *CTSW*, *KLRB1*, *KLRD1*, *KLRK1* and *NKG7*) refer to a broader cell population of granzyme-releasing cells including cytotoxic T cell and cytotoxic NK cells compared to the more specific population of ‘CD8+ T cells’ (marker genes: *CD8A* and *CD8B*). Th1 cells were significantly lower in EGFR-Ex20mut tumors compared to EGFR/ERBB2wt tumors (Figure 2D; FC = −3.3, *P* = 0.0027). Thus, EGFR-Ex20mut tumors stood out by significantly lower cytotoxic cells and Th1 cells compared to EGFR/ERBB2wt tumors.

### Gene expression analysis

**Figure 3:**
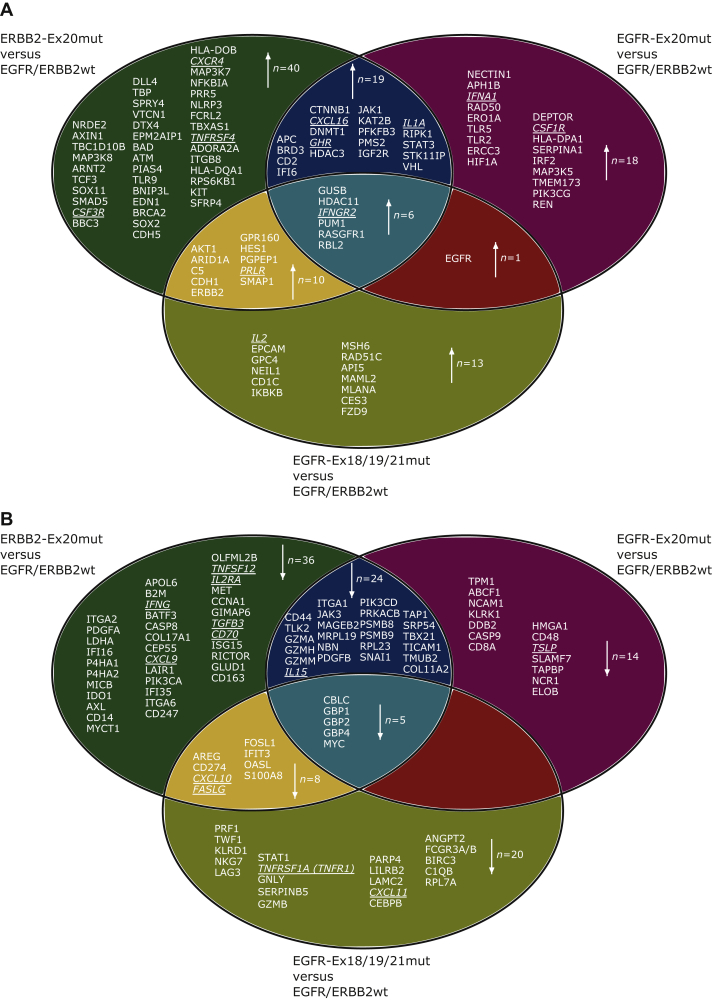
Differentially expressed genes in ERBB2-Ex20mut, EGFR-Ex20mut and EGFR-Ex18/19/21mut compared to EGFR/ERBB2wt lung adenocarcinomas. (A) Venn chart with significantly up-regulated genes. (B) Venn chart with significantly lower expressed genes. Underlined and in italics: cytokine genes.

In omnibus testing, 257 of 770 investigated genes showed significantly different expression levels in ERRB2-Ex20mut, EGFR-Ex20mut, EGFR-Ex18/19/21mut and EGFR/ERBB2wt tumors (FDR = 5%, Supplementary Table S2, available at https://doi.org/10.1016/j.esmoop.2021.100253). In detail, 148 genes were differentially expressed between ERRB2-Ex20mut and EGFR/ERBB2wt tumors, 87 genes between EGFR-Ex20mut and EGFR/ERBB2wt tumors and 63 genes between EGFR-Ex18/19/21mut and EGFR/ERBB2wt tumors. In addition, we determined these genes that are significantly up-regulated or show lower expression in this three comparison groups ERRB2-Ex20mut versus EGFR/ERBB2wt, EGFR-Ex20mut versus EGFR/ERBB2wt and EGFR-Ex18/19/21mut versus EGFR/ERBB2wt exclusively (Supplementary Table S3, available at https://doi.org/10.1016/j.esmoop.2021.100253). Forty genes were up-regulated (Figure 3A) and 36 genes showed lower expression levels (Figure 3B) in the ERRB2-Ex20mut versus EGFR/ERBB2wt group. The EGFR-Ex20mut versus EGFR/ERBB2wt group showed 18 up-regulated genes and 14 lower expressed genes. In the EGFR-Ex18/19/21mut versus EGFR/ERBB2wt group, 13 genes were up-regulated and 20 genes were lower expressed. Furthermore, we carried out for the differentially expressed genes in each comparison group a gene set enrichment analysis covering the 25 signaling pathways and functional categories annotated in this panel. Interestingly, 27.6% (8/29) of the genes with low expression in the EGFR-Ex18/19/21mut comparison group showed a significant enrichment (*P* = 0.047) for the functional category of cytotoxicity (Supplementary Table S2).

A common feature of all three comparison groups is the up-regulation of the genes *GUSB, HDAC11, IFNGR2, PUM1, RASGRF1* and *RBL2* and the lower expression of the genes *CBLC, GBP1, GBP2, GBP4* and *MYC*.

The cytolytic activity was calculated as average of granzyme A (*GZMA*) and perforin (*PRF1*) expression.^23^ The genes *GZMA* and *PRF1* showed significantly lower expression levels in the three mutated sample groups compared to the EGFR/ERBB2wt samples (Supplementary Figure S1A and B, available at https://doi.org/10.1016/j.esmoop.2021.100253).

The comparison of the ERRB2-Ex20mut and EGFR-Ex20mut samples with the EGFR/ERBB2wt samples revealed a shared group of 19 up-regulated and a group of 24 genes with lower expression levels (Figure 3A and B).

The up-regulation of the genes *AKT1, ARID1A, C5, CDH1, ERBB2, GPR160, HES1, PGPEP1, PRLR* and *SMAP1* and the lower expression levels of the genes *AREG, CD274, CXCL10, FASLG, FOSL1, IFIT3, OASL* and *S100A8* were a shared feature of the ERRB2-Ex20mut and the EGFR-Ex18/19/21mut group compared to the EGFR/ERBB2wt group.

Interestingly, comparing the two EGFR-mutated groups with the EGFR/ERBB2wt group, the *EGFR* gene is the only gene that shows different expression levels (up-regulation) in both groups, but not in the ERBB2-mutated group (Figure 3A and Supplementary Figure S1C, available at https://doi.org/10.1016/j.esmoop.2021.100253).

Compared to the EGFR/ERBB2wt samples, both the EGFR-Ex18/19/21mut and the ERRB2-Ex20mut samples, but not the EGFR-Ex20mut samples, showed a higher ERBB2 expression (Figure 3A and Supplementary Figure S1D, available at https://doi.org/10.1016/j.esmoop.2021.100253).

**Figure 4:**
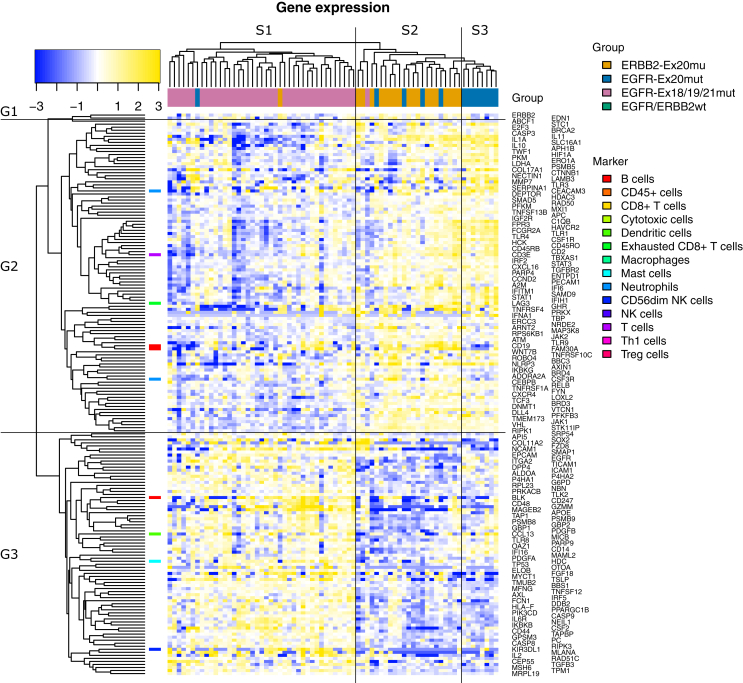
Heatmap with 185 significantly (FDR = 5%) differentially expressed genes. These gene expression levels partitioned the mutated tumor samples into three sample clusters: S1 including 95% (39/41) of EGFR-Ex18/19/21-mutated samples, S2 including 78% (18/23) of ERBB2-Ex20-mutated samples and S3 including 100% (8/8) of EGFR-Ex20-mutated samples. In these sample clusters, we detected three gene clusters (G1, G2 and G3) with different expression patterns.

To examine whether there is a difference in gene expression levels between the individual mutation groups (ERRB2-Ex20mut, EGFR-Ex20mut and EGFR-Ex18/19/21mut), a list of 185 significantly (FDR = 5%) differentially expressed genes emerged and was analyzed in a heatmap (Figure 4). This gene list partitioned the mutated tumor samples into three sample clusters: S1 including 95% (39/41) of EGFR-Ex18/19/21mut samples, S2 including 78% (18/23) of ERRB2-Ex20mut samples and S3 including 100% (8/8) of EGFR-Ex20mut samples. In these sample clusters, we detected three gene clusters (G1, G2 and G3) with different expression patterns: The sample cluster S1 with the highest proportion of EGFR-Ex18/19/21mut samples was characterized by high expression of gene cluster G3, which includes one B-cell marker (*BLK*), one marker for dendritic cells (*CCL13*), one mast cell marker (*HDC*) and one marker for CD56dim NK cells (*KIR3DL*). The sample clusters S2 and S3 with the highest proportion of ERBB2 Ex20- and EGFR Ex20-mutated samples were characterized by high expression of gene cluster G2 which includes two neutrophil markers (*CEACAM3, CSF3R*), one T-cell marker (*CD3E*), one marker for exhausted CD8+ T cells (*LAG3*) and two B-cell markers (*CD19, FAM30A*) and a low expression of gene cluster G3. For gene cluster G1, which includes the genes *ERBB2, EDN1* and *ABCF1*, we observed high expression in sample cluster S2 and an intermediate expression in sample clusters S1 and S3.

Based on this list of 185 significantly (FDR = 5%) differentially expressed genes detected by the omnibus test, we found these four most significant gene expression changes in the study cohort (Supplementary Figure S2A-D, available at https://doi.org/10.1016/j.esmoop.2021.100253): *VHL* was overexpressed in ERRB2-Ex20mut and EGFR-Ex20mut tumors compared to both EGFR-Ex18/19/21mut and EGFR/ERBB2wt tumors. *RIPK1* showed the highest expression in ERRB2-Ex20mut tumors, an intermediate expression in EGFR-Ex20mut tumors and the lowest expression in EGFR-Ex18/19/21mut and EGFR/ERBB2wt tumors. *STK11IP* showed the highest expression in ERRB2-Ex20mut tumors, an intermediate expression in EGFR-Ex20mut tumors, a lower expression in EGFR-Ex18/19/21mut tumors and the lowest expression in EGFR/ERBB2wt tumors. *JAK1* showed a similar expression pattern as *VHL*.

### Analysis of signaling pathways in cancer

In addition to estimation of immune cell populations, gene expression analysis may promote the understanding of complex mechanisms that contribute to the development and progression of cancer. Of 531 genes annotated in the KEGG map of pathways in cancer, 150 genes (28%) were covered by the used targeted expression assay. Of these, 39 genes (26%) were differentially expressed, 24 between ERRB2-Ex20mut and EGFR/ERBB2wt tumors, 7 between EGFR-Ex20mut and EGFR/ERBB2wt tumors and 10 between EGFR-Ex18/19/21mut and EGFR/ERBB2wt tumors. We assigned the changes to the map of the pathways in cancer (Supplementary Figure S3A-C, available at https://doi.org/10.1016/j.esmoop.2021.100253). Compared to EGFR/ERBB2wt tumors, we observed in mutated tumors expression changes mainly in genes related to the pathways of apoptosis, Wnt signaling and hypoxia-inducible factor 1 signaling (Supplementary Table S4A-C, available at https://doi.org/10.1016/j.esmoop.2021.100253).

### Analysis of the cytokine–cytokine receptor signaling network

Beyond the estimation of immune cell abundance in the TME, gene expression profiling offers the opportunity to gain insight into the regulation of immune response. Of 295 genes annotated in the KEGG map of cytokines and cytokine receptors, 119 genes (40%) were covered by the used targeted expression assay. Of these, 23 genes (19%) were differentially expressed, 17 between ERRB2-Ex20mut and EGFR/ERBB2wt tumors, 8 between EGFR-Ex20mut and EGFR/ERBB2wt tumors and 7 between EGFR-Ex18/19/21mut and EGFR/ERBB2wt tumors (Figure 3). Compared to EGFR/ERBB2wt tumors, four genes were differently expressed in both ERRB2-Ex20mut and EGFR-Ex20mut tumors, while nine genes showed differential expression solely in ERRB2-Ex20mut tumors and three genes showed differential expression solely in EGFR-Ex20mut tumors. Three genes were differentially expressed solely in EGFR-Ex18/19/21mut tumors compared to EGFR/ERBB2wt tumors, while no cytokine or cytokine receptor gene showed differential expression in both EGFR-Ex20mut and EGFR-Ex18/19/21mut tumors. The genes *CXCL10* and *FASLG* showed a lower expression in ERRB2-Ex20mut and EGFR-Ex18/19/21mut tumors, while *PRLR* was up-regulated compared to EGFR/ERBB2wt tumors. We assigned the changes to the map of the interaction of cytokines and receptors (Supplementary Figure S4A-C, available at https://doi.org/10.1016/j.esmoop.2021.100253).

Interestingly, the overexpression of interferon-γ receptor 2 (*IFNGR2*) was a shared feature of all three comparison groups. IFNGR2 forms the unit of the interferon-γ receptor which is needed to stimulate activation of the JAK/STAT signaling pathway by ligand binding.^24^ ERBB2-Ex20mut and EGFR-Ex20mut tumors showed, compared to EGFR/ERBB2wt tumors, an underexpression (FC = 2.5, *P* = 3.2E06 and FC = 1.9, *P* = 0.0039) of *IL15*, a cytokine that is required for NK cell development. *IL2*, an interferon that is important for the proliferation of lymphocytes, was overexpressed in EGFR-Ex18/19/21mut tumors compared to EGFR/ERBB2wt tumors (FC = 3.8, *P* = 0.00012). In EGFR-Ex20mut tumors, we observed, compared to EGFR/ERBB2wt tumors, an underexpression (FC = −3.7, *P* = 0.002) of thymic stromal lymphopoietin (*TSLP*), a growth factor that contributes to the generation of natural Treg cells in thymus.^25^ *FASLG*, a cytokine that binds to the receptor FAS that transduces apoptotic signals in cells, was underexpressed in ERRB2-Ex20mut and EGFR-Ex18/19/21mut tumors compared to EGFR/ERBB2wt tumors (FC = −3.9, *P* = 0.0018 and FC = −4.2, *P* = 6.3E−05), an observation possibly related to the lower abundance of cytotoxic cells in EGFR-positive tumors. Prolactin receptor (*PRLR*), which was suggested as a therapeutic target in subgroups of breast and of prostate cancer,^26^ was overexpressed in ERRB2-Ex20mut and EGFR-Ex18/19/21mut tumors compared to EGFR/ERBB2wt tumors (FC = 8.2, *P* = 0.00028 and FC = 4.3, *P* = 0.0012).

## Discussion

In the first part of our analysis, we detected two groups of immunologically ‘cold’ and ‘hot’ tumors, which were not restricted to one of the specific subgroups (ERRB2-Ex20mut, EGFR-Ex20mut, EGFR-Ex18/19/21mut or EGFR/ERBB2wt). However, 77% (10/13) of the EGFR-Ex20mut tumors could be assigned to the immunological group of ‘cold’ tumors compared to 65% (17/26) of the EGFR/ERBB2wt assigned to ‘hot’ tumors (*P* = 0.018). The analysis of specific cell populations revealed similar modifications of the TME in EGFR-Ex20mut and EGFR-Ex18/19/21mut compared to EGFR/ERBB2wt tumors, respectively: While we observed a lack of cytotoxic cells and Th1 cells in EGFR-Ex20mut samples, EGFR-Ex18/19/21mut samples displayed only an absence of cytotoxic cells, confirmed by gene set enrichment analysis. This suggests similar underlying mechanisms that prevent a well-balanced immune response. *EGFR*-mutant NSCLC has a unique TME^27^ which presents with both significantly reduced levels of CD8+ TIL^28^, ^29^, ^30^ and diminished CD8+ TIL function,^31^ leading to impaired cytotoxicity and resulting poor response to ICIs. In addition, EGFR-TKIs may modulate the immune response by regulating TME.^27^

Recently, it was described in *EGFR*-mutated lung cancer cells that the up-regulation of CBL proto-oncogene c (*CBLC*) contributes to tumor progression due to dysregulation of activated EGFR. In contrast, we observed a lower expression of *CBLC* in all three mutated sample groups compared to EGFR/ERBB2wt tumors. CBLC is a protein which has E3 ubiquitin ligase activity toward activated receptor tyrosine kinases. *CBLC* knockdown renders *EGFR*-mutant NSCLC cells more sensitive to TKI treatments, probably by inhibiting the transport of activated EGFR to the nucleus. Patients with NSCLC harboring *EGFR* mutations might benefit from combinational therapies with CBLC inhibition and TKI administration.^32^ In addition to *CBLC* underexpression, we found the guanylate-binding proteins *GBP1*, *GBP2* and *GBP4* were underexpressed in all three comparison groups. As lately shown by *in vitro* and *in vivo* experiments, up-regulated *GBP1* expression is associated with poor prognosis in patients with NSCLC and seems to contribute to erlotinib resistance, while decreased *GBP1* expression seems to have the opposite effect. Functional testing confirmed that *GBP1* regulates epithelial–mesenchymal transition in NSCLC.^33^ Recently, it was shown that *GBP1* and *GBP4* are IFNG-dependent and were directly co-expressed with *CD8A* in colorectal cancer. In lung squamous carcinoma, up-regulated *IFNG* correlates with up-regulation of *GBP1* and *GBP4*.^34^ Furthermore, we observed underexpression of cellular oncogene *MYC* in all three mutated sample groups compared to EGFR/ERBB2wt tumors. These results match the results of a more recent study that revealed an association of *MYC* overexpression with the histological subtype in lung cancer: In ADC, *MYC* expression was normal, while in squamous cell carcinoma *MYC* overexpression was present in >50% of tested samples.^35^

The up-regulation of the genes *RBL2, PUM1, IFNGR2, HDAC11, GUSB* and *RASGRF1* is a shared feature of all three comparison groups in our cohort. The retinoblastoma-like protein *RBL2*, a key factor in cell cycle regulation and apoptosis, was lately identified as a direct substrate of the AKT kinase which is known as a key antiapoptotic factor that is hyperactive in multiple cancer types. *AKT* inhibition increased *RBL2* expression and triggered apoptosis in both lung cancer and mesothelioma cell lines.^36^ MicroRNA (miRNA) plays a major role in the biological behavior of cancer cells by regulating the expression of target genes. Most recently, it was reported that *PUM1* could be the target of miR-411-5p, for which overexpression may inhibit proliferation and promote apoptosis of NSCLC cells.^37^ Recent studies demonstrated that lung ADC cells showed IFNG hypo-responsiveness even though there were no differences in the expression of *IFNGR1* and *IFNGR2.*^38^ Our findings of overexpression of histone deacetylase *HDAC11* correspond to those of a recent study which reports that high *HDAC11* levels in human lung tumor tissues correlate with poor prognosis. Inhibition of *HDAC11* not only significantly reduces self-renewal capacity of cancer stem cells from NSCLC but also decreases *SOX2* expression that is essential for their maintenance.^39^

Patients with NSCLC with *EGFR* exon 20 insertions show very poor response rates to ICIs, especially when given in the first-line therapy setting.^40^ This could indicate that these tumors have a more immunosuppressed microenvironment than, for example, ERBB2 Ex20-mutated tumors. In our cohort, EGFR-Ex20mut tumors stood out by significantly lower cytotoxic cells and Th1 cells, while ERRB2-Ex20mut samples exhibited a lack of CD56dim NK cells. Furthermore, we observed in ERRB2-Ex20mut samples a significant up-regulation of *TNFRSF4* (also known as *OX40*), a costimulatory molecule which modifies T-cell response,^41^ and *CXCR4*, which is an alpha-chemokine receptor specific for stromal-derived-factor-1 (SDF-1 also known as CXCL12), a molecule endowed with potent chemotactic activity for lymphocytes.^42^ In a phase I study of patients with refractory metastatic solid tumors, administration of a murine agonistic anti-human OX-40 monoclonal antibody resulted in an increased proliferation of CD8+ and CD4+FoxP3−T cells, thus restoring dendritic cell and antitumor activity.^43^ It was shown that many NSCLC cell lines express high levels of *CXCR4* and that SDF-1-activated *CXCR4* promotes migration and invasion of these cell lines *in vitro*.^44^ Furthermore, we observed a significant underexpression for two molecules belonging to the tumor necrosis factor (TNF) superfamily: the cytokine *TNFSF12* (aka *TWEAK*), which can induce apoptosis via multiple pathways of cell death in a cell type-specific manner, and for *CD70*, which is expressed on highly activated lymphocytes.^43^ Lower expression levels of *TWEAK*, respectively, *CD70* are described in NSCLC, especially for tumors carrying activating *EGFR* mutations.^45^^,^^46^ Additionally, we detected a significant down-regulation of *IFNG.* This observation might be related to the lower abundance of CD56dim NK cells in ERRB2-Ex20mut tumors. Mature (CD56dim) NK cells are generally considered more cytotoxic and carry out antibody-dependent cell-mediated cytotoxicity, whereas immature (CD56bright) NK cells are effective producers of IFNG, which is conventionally recognized as an inflammatory cytokine that plays a central role in antitumor immunity.^47^ Low levels of *IFNG* in the TME increase the risk of tumor metastasis during immunotherapy and are closely associated with poor prognosis in patients with NSCLC.^48^ The findings of a recent phase Ib trial showed that the administration of nivolumab in combination with an IL15 superagonist led to expansion of NK cells and CD8+ T cells as well as raised serum concentration of IFNG in patients with NSCLC.^49^

In EGFR-Ex20mut samples, we observed significant up-regulation of *IFNA* and *CSF1R*. Recent data demonstrated that upon T-cell receptor recognition, specific NSCLC tumor cells strongly induced the expression of TNFSF10, an apoptosis-inducing cytokine, on CD4+ but not on CD8+ cytotoxic T-cell clones. This expression was slightly increased in the presence of the immune modulating cytokine IFN-α, leading to tumor growth inhibition.^50^ *CSF1R*, a receptor for colony stimulating factor 1, is overexpressed in many cancers.^51^

Interestingly, the proinflammatory cytokine *IL2* was up-regulated for EGFR-Ex18/19/21mut samples, while the chemokine *CXCL11* and TNF receptor superfamily member 1A *TNFRSF1A* (aka *TNFR1*) were lower expressed. *IL2* is known to modulate the development and expansion of regulatory T cells exerting immunosuppressive effects.^52^ EGFR-positive tumors feature a CD8+-deprived environment that is modulated by lower expression levels of *CXCL11*, the ligand for CXCR3 on cytotoxic T cells, negatively modulating CD8+ T-cell migration.^53^^,^^54^ *TNFR1* is one of the major receptors for the TNF-α, which mediates apoptosis and regulates inflammation. It was described that MEK inhibition leads to increased cell surface expression of TNFR1 and may sensitize tumor cells to TNFA-induced apoptosis. This finding suggests that therapies that enhance cytokine production in the TME (e.g. ICI) may synergize with MEK inhibitors.^55^

It was shown that protein expression levels of chemokine CXCL16 which regulates inflammation, growth hormone receptor (GHR) and PRL were elevated in lung tumor tissue, which was associated with decreased survival of patients with lung cancer.^56^^,^^57^ We observed an up-regulation of *CXCL16,* a chemokine, playing an important role in inflammatory regulation, and *GHR* in ERRB2-Ex20mut and EGFR-Ex20mut tumors. The results of a recent study suggested that extracellular PRL enhanced NSCLC cell proliferation and promoted *JAK2/STAT3* signaling activity through GHR, but not PRLR as previously reported in breast and prostate cancers.^26^^,^^57^ The regulation of autocrine *PRL* and *GHR* levels might evolve into a therapeutic strategy in patients with NSCLC.

Lately, two novel and irreversibly binding TKIs were tested in clinical trials: mobocertinib, which binds to *EGFR* via covalent modification of the Cys797 residue in the *EGFR* active site,^58^ and poziotinib, a covalent and potent inhibitor of *EGFR* and *ERBB2* exon 20 insertions.^59^ Despite encouraging earlier results for the efficacy of poziotinib, the Zenith 20 trial revealed a low response rate of 14% in patients with NSCLC with *EGFR* exon 20 insertions.^9^ Moreover, both TKIs showed high rates of *EGFR* wild-type-driven toxicity, limiting their clinical applicability.^2^ Recently, amivantamab, an EGFR-MET bispecific antibody with immune cell-directing activity demonstrated a response rate of 40% with good tolerability in pretreated patients with NSCLC harboring *EGFR* exon 20 insertions.^60^^,^^61^ To date, no targeted therapies are approved for patients with NSCLC with *EGFR* or *ERBB2* exon 20-activating mutations, which presents an unmet clinical need.

In conclusion, our data revealed heterogeneous types of TME modification in *ERBB2*-positive and *EGFR*-positive NSCLC, respectively, each accompanied by a specific pattern of cytokine signaling. Given this complexity, it is essential to identify the optimal sequence of treatment and strategies for patients with NSCLC with *ERBB2/EGFR* mutations. Moreover, mechanisms to induce long-lasting antitumor activity in the TME and to maximize the effect of immunotherapy in patients can still be improved. This may be achievable by tailored combinations of ICIs with radio- and chemotherapy, or by more subtle, specific approaches that either inhibit specific immunosuppressive agents enriched in or supplement and boost proinflammatory molecules depleted in ‘cold’ tumors. There is also a clear unmet need for establishing prognostic molecular and clinical markers, dosages, schedules, the optimal sequence of treatment and strategies when combining immunotherapy with other therapies. There is a substantial need to investigate the variety of immune reactivity in oncogene-addicted NSCLC, leading to the detection of immunomodulators currently explored in various pre-clinical studies and clinical trials.
