# Supplementary material for: The immune microenvironment in EGFR- and ERBB2-mutated lung adenocarcinoma
Source: ESMO Open. 2021 Sep 3;6(5):100253. doi: 10.1016/j.esmoop.2021.100253 (PMC8426209; doi:10.1016/j.esmoop.2021.100253)
Supplement: Supplemental Data 3 [file mmc3.docx]

**Supplementary Table S3: Down- and upregulated genes in ERBB2-Ex20mut, EGFR-Ex20mut and EGFR-Ex18/19/21mut tumors.**

|  | ***ERBB2-Ex20mut – downregulated genes*** | | | | | | | |
| --- | --- | --- | --- | --- | --- | --- | --- | --- |
| ***Gene*** | ***Name*** | | ***General Annotation*** | | ***Gene is assigned to*** | | | |
|  |  | |  | | ***Tumor cells*** | | ***Microenvironment*** | |
| *ITGA2* | Integrin subunit alpha 2 | | Immune Cell Adhesion and Migration; Matrix Remodeling and Metastasis; PI3K-Akt | |  | |  | |
| *PDGFA* | Platelet derived growth factor subunit A | | Matrix Remodeling and Metastasis; PI3K-Akt; MAPK | | Cell proliferation | |  | |
| *LDHA* | Lactate dehydrogenase A | | Metabolic Stress; Hypoxia | | Altered glycolytic metabolism | |  | |
| *IFI16* | Interferon gamma inducible protein 16 | | Interferon Signaling; Cytotoxicity | |  | |  | |
| *P4HA1* | Prolyl 4-hydroxylase subunit alpha 1 | | Endoplasmic reticulum | |  | |  | |
| *P4HA2* | Prolyl 4-hydroxylase subunit alpha 2 | | Endoplasmic reticulum | |  | |  | |
| *MICB* | MHC class I polypeptide-related sequence B | | Immune response-activating cell surface receptor signaling pathway | |  | |  | |
| *IDO1* | Indoleamine 2,3-dioxygenase 1 | | Lymphoid Compartment | |  | |  | |
| *AXL* | AXL receptor tyrosine kinase | | Positive regulation of cytokine-mediated signaling pathway | |  | |  | |
| *CD14* | CD14 molecule | | Apoptosis; MAPK; Myeloid Compartment | |  | | Monocytes, macrophages | |
| *MYCT1* | MYC target 1 | | Nucleoplasm | | Regulation of MYC target genes | |  | |
| *APOL6* | Apolipoprotein L6 | | Lipid transport | |  | |  | |
| *B2M* | Beta-2-microglobulin | | Antigen Presentation; Interferon Signaling | |  | |  | |
| *IFNG* | Interferon gamma | | Antigen Presentation; Interferon Signaling; JAK-STAT Signaling; Cytotoxicity; Hypoxia; TGF-beta Signaling; Lymphoid Compartment | |  | | Activated lymphocytes | |
| *BATF3* | Basic leucine zipper ATF-like transcription factor 3 | | Antigen Presentation | |  | |  | |
| *CASP8* | Caspase 8 | | Apoptosis | |  | |  | |
| *COL17A1* | Collagen type XVII alpha 1 chain | | Matrix Remodeling and Metastasis; Myeloid Compartment | |  | |  | |
| *CEP55* | Centrosomal protein 55 | | Cell Proliferation | |  | |  | |
| *CXCL9* | C-X-C motif chemokine ligand 9 | | Cytokine and Chemokine Signaling; Lymphoid Compartment | |  | |  | |
| *LAIR1* | Leukocyte associated immunoglobulin like receptor 1 | | Neutrophil degranulation | |  | |  | |
| *PIK3CA* | Phosphatidylinositol-4,5-bisphosphate 3-kinase catalytic subunit alpha | | Costimulatory Signaling; Cytokine and Chemokine Signaling; Immune Cell Adhesion and Migration; JAK-STAT Signaling; Autophagy; Metabolic Stress; Hypoxia; PI3K-Akt; MAPK | | Activation of AKT1 | |  | |
| *IFI35* | Interferon induced protein 35 | | Interferon Signaling; Cytotoxicity | |  | |  | |
| *OLFML2B* | Olfactomedin like 2B | | Extracellular region | |  | |  | |
| *TNFSF12* | TNF superfamily member 12 | | NF-kappaB Signaling | |  | |  | |
| *IL2RA* | Interleukin 2 receptor subunit alpha | | Costimulatory Signaling; Cytokine and Chemokine Signaling; JAK-STAT Signaling; PI3K-Akt | |  | |  | |
| *MET* | MET proto-oncogene, receptor tyrosine kinase | | Metabolic Stress; PI3K-Akt; MAPK | | Receptor tyrosine kinase | |  | |
| *CCNA1* | Cyclin A1 | | DNA Damage Repair; Cell Proliferation; Metabolic Stress | |  | |  | |
| *GIMAP6* | GTPase, IMAP family member 6 | | Nucleoplasm | |  | |  | |
| *TGFB3* | Transforming growth factor beta 3 | | Matrix Remodeling and Metastasis; TGF-beta Signaling; MAPK | |  | |  | |
| *CD70* | CD70 molecule | | Costimulatory Signaling; NF-kappaB Signaling; Lymphoid Compartment | |  | | Activated T and B cells | |
| *ISG15* | ISG15 ubiquitin like modifier | | DNA Damage Repair; Interferon Signaling; Cytotoxicity; Lymphoid Compartment | |  | |  | |
| *RICTOR* | RPTOR independent companion of MTOR complex 2 | | Costimulatory Signaling; Metabolic Stress | |  | |  | |
| *ITGA6* | Integrin subunit alpha 6 | | Immune Cell Adhesion and Migration; Matrix Remodeling and Metastasis; PI3K-Akt | |  | |  | |
| *CD247* | CD247 molecule | | Costimulatory Signaling | |  | | T cells | |
| *GLUD1* | Glutamate dehydrogenase 1 | | Glutamate biosynthetic process | |  | |  | |
| *CD163* | CD163 molecule | | Acute-phase response | |  | | Macrophages | |
|  | ***EGFR-Ex20mut – downregulated genes*** | | | | | | | |
| ***Gene*** | ***Name*** | | ***General Annotation*** | | ***Gene is assigned to*** | | | |
|  |  | |  | | ***Tumor cells*** | | ***Microenvironment*** | |
| *TPM1* | Tropomyosin 1 | | Angiogenesis | |  | |  | |
| *ABCF1* | ATP binding cassette subfamily F member 1 | | Inflammatory response | |  | |  | |
| *NCAM1* | Neural cell adhesion molecule 1 | | Immune Cell Adhesion and Migration; Matrix Remodeling and Metastasis; Interferon Signaling | |  | |  | |
| *KLRK1* | Killer cell lectin like receptor K1 | | Cytotoxicity; Lymphoid Compartment | |  | | Cytotoxic cells | |
| *DDB2* | Damage specific DNA binding protein 2 | | DNA Damage Repair | |  | |  | |
| *CASP9* | Caspase 9 | | Apoptosis; PI3K-Akt | |  | |  | |
| *CD8A* | CD8a molecule | | Antigen Presentation; Immune Cell Adhesion and Migration; Lymphoid Compartment | |  | | CD8 T cells | |
| *HMGA1* | High mobility group AT-hook 1 | | Epigenetic Regulation; Metabolic Stress | |  | |  | |
| *CD48* | CD48 molecule | | Costimulatory Signaling; Lymphoid Compartment | |  | |  | |
| *TSLP* | Thymic stromal lymphopoietin | | Costimulatory Signaling; JAK-STAT Signaling | |  | |  | |
| *SLAMF7* | SLAM family member 7 | | Lymphoid Compartment | |  | |  | |
| *TAPBP* | TAP binding protein | | Antigen Presentation | |  | |  | |
| *NCR1* | Natural cytotoxicity triggering receptor 1 | | Natural killer cell activation | |  | | NK cells | |
| *ELOB* | Elongin B | | Transcription elongation from RNA polymerase II promoter | |  | |  | |
|  | ***EGFR-Ex18/19/21mut – downregulated genes*** | | | | | | | |
| ***Gene*** | ***Name*** | | ***General Annotation*** | ***Gene is assigned to*** | | | | |
|  |  | |  | ***Tumor cells*** | | | | ***Microenvironment*** |
| *PRF1* | Perforin 1 | | Cytotoxicity; Lymphoid Compartment |  | | | | Cytotoxic cells |
| *TWF1* | Twinfilin actin binding protein 1 | | Regulation of actin phosphorylation |  | | | |  |
| *KLRD1* | Killer cell lectin like receptor D1 | | Antigen Presentation; Cytotoxicity; Lymphoid Compartment |  | | | | Cytotoxic cells |
| *NKG7* | Natural killer cell granule protein 7 | | Protein binding |  | | | | Cytotoxic cells |
| *LAG3* | Lymphocyte activating 3 | | Costimulatory Signaling; Lymphoid Compartment |  | | | | Exhausted CD8 |
| *STAT1* | Signal transducer and activator of transcription 1 | | Cytokine and Chemokine Signaling; Interferon Signaling; JAK-STAT Signaling; Cytotoxicity; Lymphoid Compartment |  | | | |  |
| *TNFRSF1A* | TNF receptor superfamily member 1A | | NF-kappaB Signaling; MAPK |  | | | |  |
| *GNLY* | Granulysin | | Cytotoxicity; Lymphoid Compartment |  | | | | Cytotoxic cells |
| *SERPINB5* | Serpin family B member 5 | | Angiogenesis |  | | | |  |
| *GZMB* | Granzyme B | | Cytotoxicity; Apoptosis; Notch Signaling; Lymphoid Compartment |  | | | | Cytotoxic cells |
| *PARP4* | Poly (ADP-ribose) polymerase family member 4 | | DNA Damage Repair |  | | | |  |
| *LILRB2* | Leukocyte immunoglobulin like receptor B2 | | Costimulatory Signaling; Myeloid Compartment |  | | | |  |
| *LAMC2* | Laminin subunit gamma 2 | | Matrix Remodeling and Metastasis; PI3K-Akt |  | | | |  |
| *CXCL11* | C-X-C motif chemokine ligand 11 | | Cytokine and Chemokine Signaling; Lymphoid Compartment |  | | | |  |
| *CEBPB* | CCAAT enhancer binding protein beta | | Metabolic Stress; Myeloid Compartment |  | | | |  |
| *ANGPT2* | Angiopoietin 2 | | Angiogenesis; Hypoxia; PI3K-Akt; MAPK |  | | | |  |
| *FCGR3A/B* | Fc fragment of IgG receptor IIIa | | Regulation of immune response |  | | | | Neutrophils |
| *BIRC3* | Baculoviral IAP repeat containing 3 | | Apoptosis; NF-kappaB Signaling |  | | | |  |
| *C1QB* | Complement C1q B chain | | Complement activation |  | | | |  |
| *RPL7A* | Ribosomal protein L7a | | Angiogenesis |  | | | |  |
|  | ***ERBB2-Ex20mut – upregulated genes*** | | | | | | | |
| ***Gene*** | ***Name*** | | ***General Annotation*** | | ***Gene is assigned to*** | | | |
|  |  | |  | | ***Tumor cells*** | | ***Microenvironment*** | |
| *NRDE2* | NRDE-2, necessary for RNA interference, domain containing | | Biological process | |  | |  | |
| *AXIN1* | Axin 1 | | Wnt Signaling | | Wnt Signaling | |  | |
| *TBC1D10B* | TBC1 domain family member 10B | | Regulation of GTPase activity | |  | |  | |
| *MAP3K8* | Mitogen-activated protein kinase kinase kinase 8 | | Costimulatory Signaling; MAPK | |  | |  | |
| *ARNT2* | Aryl hydrocarbon receptor nuclear translocator 2 | | Response to hypoxia | |  | |  | |
| *TCF3* | Transcription factor 3 | | Negative regulation of transcription | |  | |  | |
| *SOX11* | SRY-box 11 | | Wnt Signaling | | Wnt Signaling | |  | |
| *SMAD5* | SMAD family member 5 | | TGF-beta Signaling | |  | |  | |
| *CSF3R* | Colony stimulating factor 3 receptor | | Cytokine and Chemokine Signaling; JAK-STAT Signaling; PI3K-Akt; Myeloid Compartment | |  | | Neutrophils | |
| *BBC3* | BCL2 binding component 3 | | Cytotoxicity; Apoptosis | |  | |  | |
| *DLL4* | Delta like canonical Notch ligand 4 | | Angiogenesis; Notch Signaling; Myeloid Compartment | |  | |  | |
| *TBP* | TATA-box binding protein | | Regulation of transcription | |  | |  | |
| *SPRY4* | Sprouty RTK signaling antagonist 4 | | Cytotoxicity | |  | |  | |
| *VTCN1* | V-set domain containing T cell activation inhibitor 1 | | Costimulatory Signaling; Immune Cell Adhesion and Migration | |  | |  | |
| *DTX4* | Deltex E3 ubiquitin ligase 4 | | Notch Signaling | |  | |  | |
| *EPM2AIP1* | EPM2A interacting protein 1 | | Protein binding | |  | |  | |
| *BAD* | BCL2 associated agonist of cell death | | Apoptosis; Autophagy; PI3K-Akt; MAPK | |  | |  | |
| *ATM* | ATM serine/threonine kinase | | DNA Damage Repair; Cell Proliferation; Metabolic Stress | |  | |  | |
| *PIAS4* | Protein inhibitor of activated STAT 4 | | DNA Damage Repair; JAK-STAT Signaling; Cell Proliferation | |  | |  | |
| *TLR9* | Toll like receptor 9 | | Inflammatory response | |  | |  | |
| *HLA-DOB* | Major histocompatibility complex, class II, DO beta | | Antigen Presentation; Immune Cell Adhesion and Migration; Lymphoid Compartment | | |  |  | |
| *CXCR4* | C-X-C motif chemokine receptor 4 | | Cytokine and Chemokine Signaling; Immune Cell Adhesion and Migration | | |  |  | |
| *MAP3K7* | Mitogen-activated protein kinase kinase kinase 7 | | Costimulatory Signaling; Autophagy; Wnt Signaling; MAPK | | | Wnt Signaling |  | |
| *NFKBIA* | NFKB inhibitor alpha | | Costimulatory Signaling; Cytokine and Chemokine Signaling; NF-kappaB Signaling | | |  |  | |
| *PRR5* | Proline rich 5 | | Costimulatory Signaling; Metabolic Stress | | |  |  | |
| *NLRP3* | NLR family pyrin domain containing 3 | | Myeloid Compartment | | |  |  | |
| *FCRL2* | Fc receptor like 2 | | Cell-cell signaling | | |  | B cells | |
| *TBXAS1* | Thromboxane A synthase 1 | | Cyclooxygenase pathway | | |  |  | |
| *TNFRSF4* | TNF receptor superfamily member 4 | | NF-kappaB Signaling | | |  |  | |
| *ADORA2A* | Adenosine A2a receptor | | Costimulatory Signaling | | |  |  | |
| *ITGB8* | Integrin subunit beta 8 | | Immune Cell Adhesion and Migration; Matrix Remodeling and Metastasis; PI3K-Akt | | |  |  | |
| *HLA-DQA1* | Major histocompatibility complex, class II, DQ alpha 1 | | Antigen Presentation; Costimulatory Signaling; Immune Cell Adhesion and Migration; Interferon Signaling | | |  |  | |
| *RPS6KB1* | Ribosomal protein S6 kinase B1 | | Autophagy; Metabolic Stress; Hypoxia; TGF-beta Signaling; PI3K-Akt | | |  |  | |
| *KIT* | KIT proto-oncogene, receptor tyrosine kinase | | Metabolic Stress; PI3K-Akt; MAPK | | |  |  | |
| *SFRP4* | Secreted frizzled related protein 4 | | Wnt Signaling | | | Wnt Signaling |  | |
| *BNIP3L* | BCL2 interacting protein 3 like | | Regulation of apoptotic process | | |  |  | |
| *EDN1* | Endothelin 1 | | Angiogenesis; Hypoxia | | |  |  | |
| *BRCA2* | BRCA2 DNA repair associated | | DNA Damage Repair; Cell Proliferation | | |  |  | |
| *SOX2* | SRY-box 2 | | Wnt Signaling | | | Wnt Signaling |  | |
| *CDH5* | Cadherin 5 | | Immune Cell Adhesion and Migration | | |  |  | |
|  | ***EGFR-Ex20mut – upregulated genes*** | | | | | | | |
| ***Gene*** | ***Name*** | ***General Annotation*** | | | | ***Gene is assigned to*** | | |
|  |  |  | | | | ***Tumor cells*** | ***Microenvironment*** | |
| *NECTIN1* | Nectin cell adhesion molecule 1 | Immune Cell Adhesion and Migration | | | |  |  | |
| *APH1B* | Aph-1 homolog B, gamma-secretase subunit | Notch Signaling | | | |  |  | |
| *IFNA1* | Interferon alpha 1 | Interferon Signaling; JAK-STAT Signaling; PI3K-Akt | | | |  |  | |
| *RAD50* | RAD50 double strand break repair protein | DNA Damage Repair; Cell Proliferation; Metabolic Stress | | | |  |  | |
| *ERO1A* | Endoplasmic reticulum oxidoreductase 1 alpha | Metabolic Stress | | | |  |  | |
| *TLR5* | Toll like receptor 5 | Inflammatory response | | | |  |  | |
| *TLR2* | Toll like receptor 2 | PI3K-Akt; Myeloid Compartment | | | |  |  | |
| *ERCC3* | ERCC excision repair 3, TFIIH core complex helicase subunit | DNA Damage Repair | | | |  |  | |
| *HIF1A* | Hypoxia inducible factor 1 subunit alpha | Autophagy; Metabolic Stress; Hypoxia; Notch Signaling | | | |  |  | |
| *DEPTOR* | DEP domain containing MTOR interacting protein | Autophagy; Metabolic Stress | | | |  |  | |
| *CSF1R* | Colony stimulating factor 1 receptor | Cytokine and Chemokine Signaling; PI3K-Akt; MAPK; Myeloid Compartment | | | |  |  | |
| *HLA-DPA1* | Major histocompatibility complex, class II, DP alpha 1 | Antigen Presentation; Costimulatory Signaling; Immune Cell Adhesion and Migration; Interferon Signaling | | | |  |  | |
| *SERPINA1* | Serpin family A member 1 | Myeloid Compartment | | | |  |  | |
| *IRF2* | Interferon regulatory factor 2 | Interferon Signaling | | | |  |  | |
| *MAP3K5* | Mitogen-activated protein kinase kinase kinase 5 | Metabolic Stress; MAPK | | | |  |  | |
| *TMEM173* | Transmembrane protein 173 | Activation of innate immune response | | | |  |  | |
| *PIK3CG* | Phosphatidylinositol-4,5-bisphosphate 3-kinase catalytic subunit gamma | Cytokine and Chemokine Signaling; Immune Cell Adhesion and Migration; JAK-STAT Signaling; Metabolic Stress; Hypoxia; PI3K-Akt; MAPK | | | |  |  | |
| REN | Renin | Response to lipopolysaccharide | | | |  |  | |
|  | ***EGFR Ex18/19/21mut – upregulated genes*** | | | | | | | |
| ***Gene*** | ***Name*** | ***General Annotation*** | | | | ***Gene is assigned to*** | | |
|  |  |  | | | | ***Tumor cells*** | | ***Microenvironment*** |
| *IL2* | Interleukin 2 | Costimulatory Signaling; Cytokine and Chemokine Signaling; JAK-STAT Signaling; PI3K-Akt | | | |  | |  |
| *EPCAM* | Epithelial cell adhesion molecule | Positive regulation of cell proliferation | | | |  | |  |
| *GPC4* | Glypican 4 | Wnt Signaling | | | |  | |  |
| *NEIL1* | Nei like DNA glycosylase 1 | DNA Damage Repair | | | |  | |  |
| *CD1C* | CD1c molecule | Antigen Presentation; Lymphoid Compartment | | | |  | |  |
| *IKBKB* | Inhibitor of nuclear factor kappa B kinase subunit beta | Costimulatory Signaling; Cytokine and Chemokine Signaling; Metabolic Stress; NF-kappaB Signaling; PI3K-Akt; MAPK | | | |  | |  |
| *MSH6* | MutS homolog 6 | DNA Damage Repair | | | |  | |  |
| *RAD51C* | RAD51 paralog C | DNA Damage Repair; Cell Proliferation | | | |  | |  |
| *API5* | Apoptosis inhibitor 5 | Apoptotic process | | | |  | |  |
| *MAML2* | CREB regulated transcription coactivator 1 | Notch Signaling | | | |  | |  |
| *MLANA* | Melan-A | Melanosome | | | |  | |  |
| *CES3* | Carboxylesterase 3 | Angiogenesis | | | |  | |  |
| *FZD9* | Frizzled class receptor 9 | Wnt Signaling | | | |  | |  |
