# Supplementary material for: The immune microenvironment in EGFR- and ERBB2-mutated lung adenocarcinoma
Source: ESMO Open. 2021 Sep 3;6(5):100253. doi: 10.1016/j.esmoop.2021.100253 (PMC8426209; doi:10.1016/j.esmoop.2021.100253)
Supplement: Supplemental Data 4-8 [file mmc4.pdf]

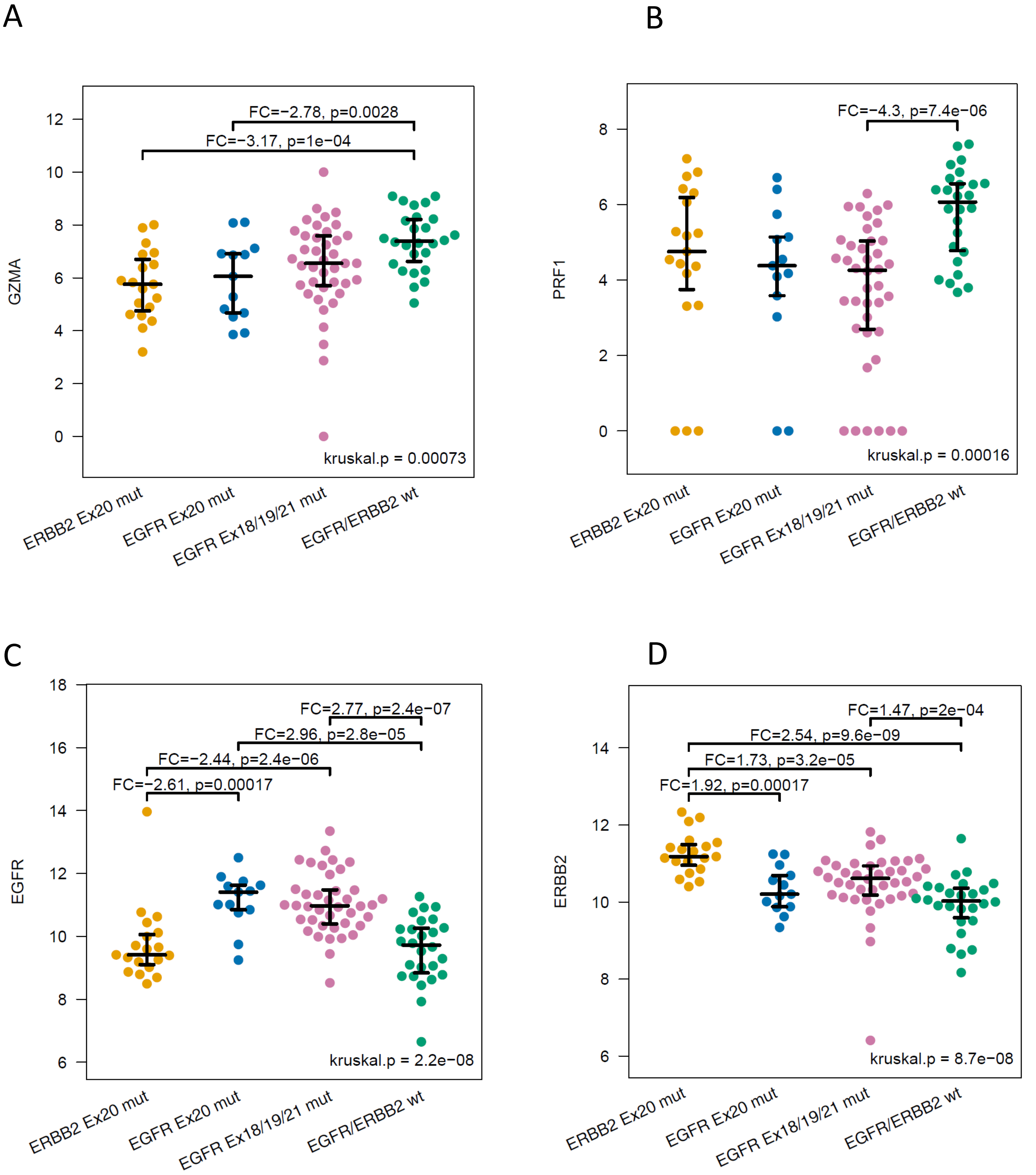

Suppl. Figure S1: Differential gene expression between ERBB2-Ex20mut, EGFR-Ex20mut, EGFR-Ex18/19/21mut, and EGFR/ERBB2wt lung adenocarcinoma. **A** The gene *GZMA* showed significantly lower expression in ERBB2-Ex20mut, EGFR-Ex20mut and EGFR-Ex18/19/21mut tumors compared to the EGFR/ERBB2wt tumors. **B** The gene *PRF1* showed significantly lower expression in ERBB2-Ex20mut, EGFR-Ex20mut and EGFR-Ex18/19/21mut tumors compared to the EGFR/ERBB2wt tumors. **C** Compared to ERBB2-Ex20mut and EGFR/ERBB2wt tumors *EGFR* is upregulated in EGFR-Ex20mut and EGFR-Ex18/19/21mut tumors. **D** EGFR-Ex18/19/21mut samples and ERBB2-Ex20mut tumors showed a higher *ERBB2* expression compared to EGFR/ERBB2wt and EGFR-Ex20mut tumors.

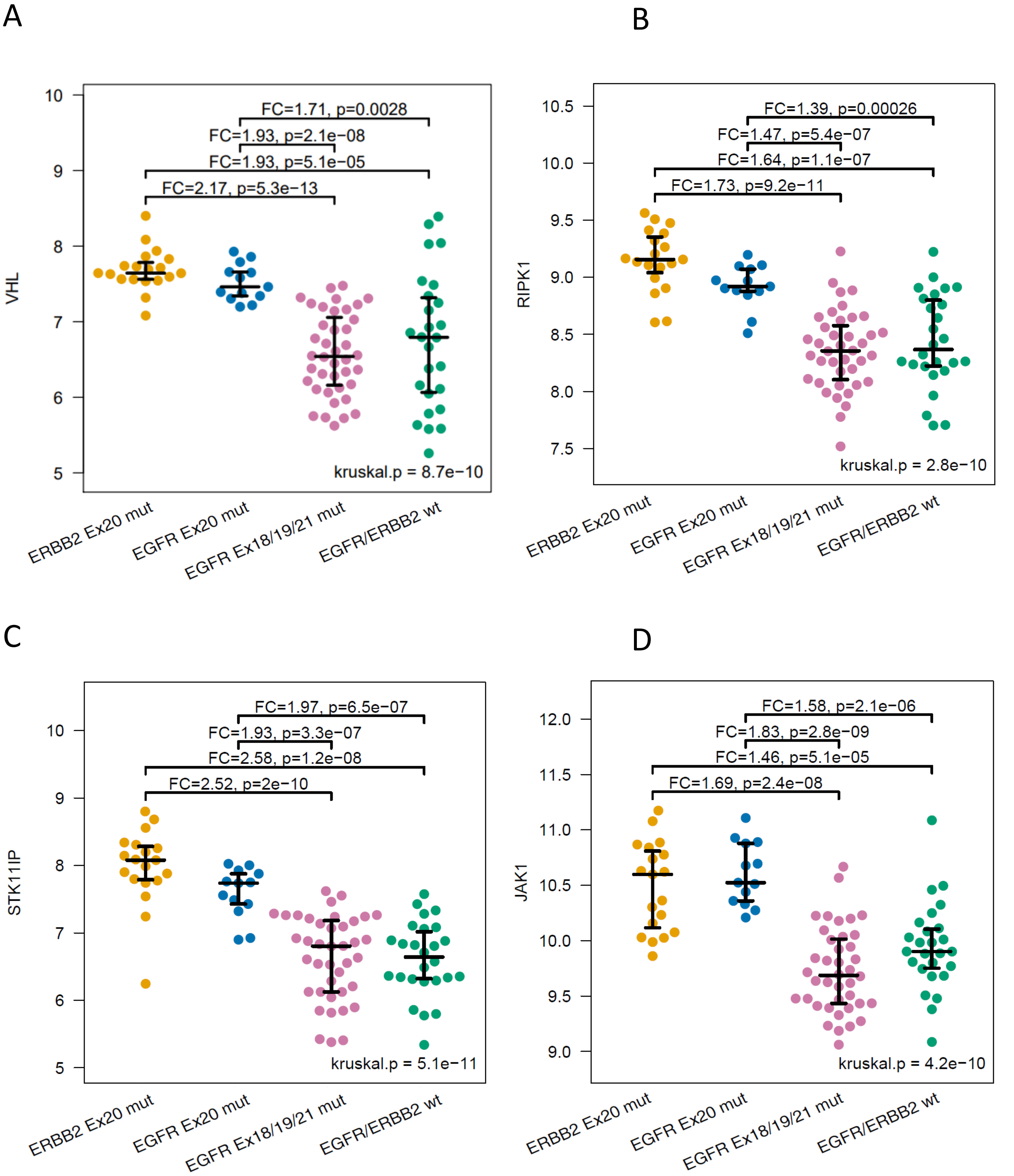

Suppl. Figure S2: Differential gene expression between ERBB2-Ex20mut, EGFR-Ex20mut, EGFR-Ex18/19/21mut, and EGFR/ERBB2wt lung adenocarcinomas **A-D** Expression levels of most significantly differentially expressed genes in omnibus testing: **A** *VHL* was overexpressed in ERBB2-Ex20mut and EGFR-Ex20mut compared to both EGFR-Ex18/19/21mut tumors and EGFR/ERBB2wt tumors. **B** *RIPK1* showed the highest expression in ERBB2-Ex20mut tumors, an intermediate expression in EGFR-Ex20mut tumors, and the lowest expression in EGFR-Ex18/19/21mut tumors and in EGFR/ERBB2wt tumors. **C** *STK11IP* showed the highest expression in ERBB2-Ex20mut tumors, an intermediate expression in EGFR-Ex20mut tumors, a lower expression in EGFR-Ex18/19/21mut tumors and the lowest expression in EGFR/ERBB2wt tumors. **D** *JAK1* showed a similar expression pattern as *VHL*.

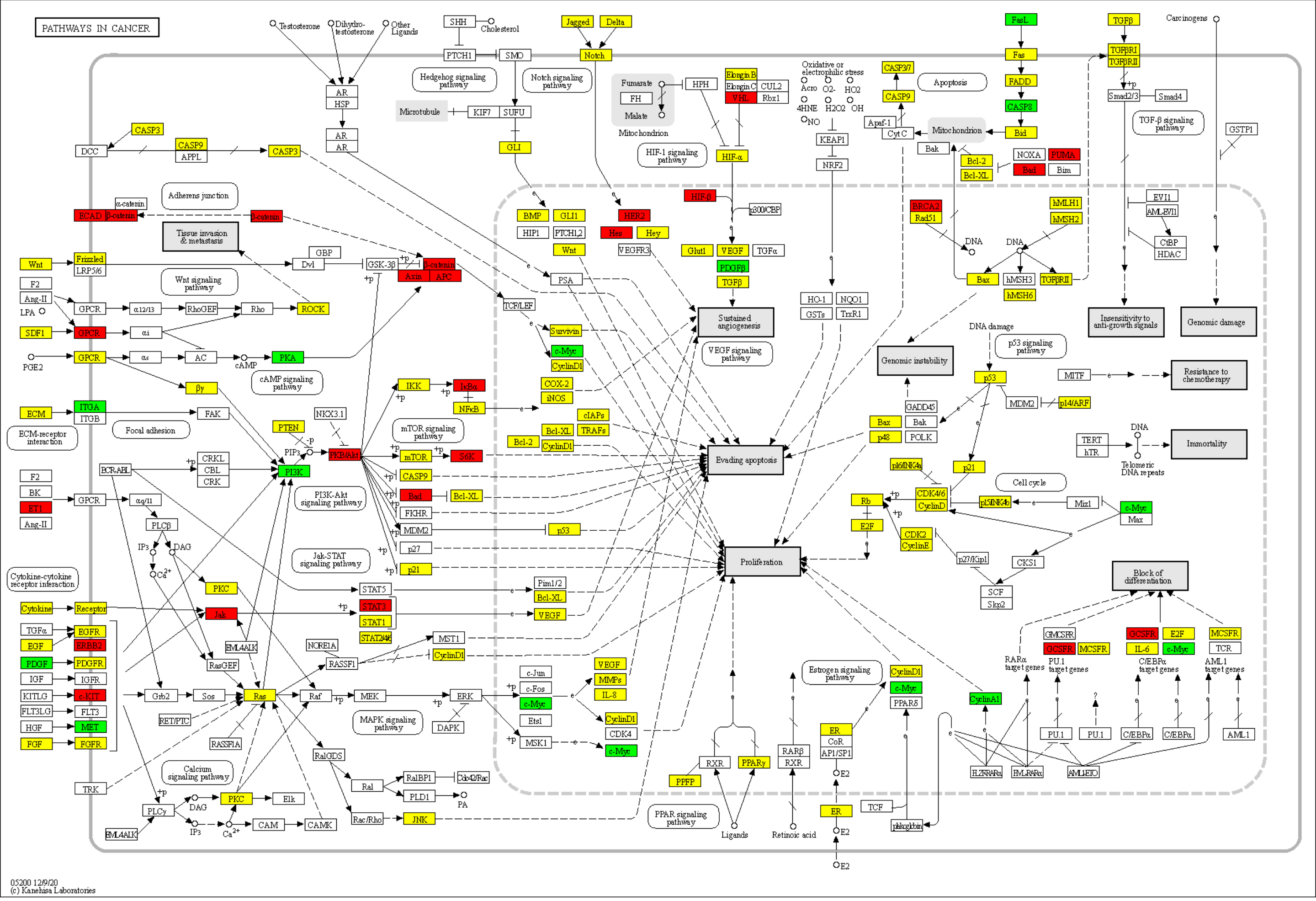

Supplementary Figure S3 A: Gene expression changes between ERBB2-Ex20mut and EGFR/ERBB2wt tumors in the pathways in cancer system. Red = overexpression, green = underexpression, yellow = no significant change, white = human gene, but not covered by the targeted gene expression assay.

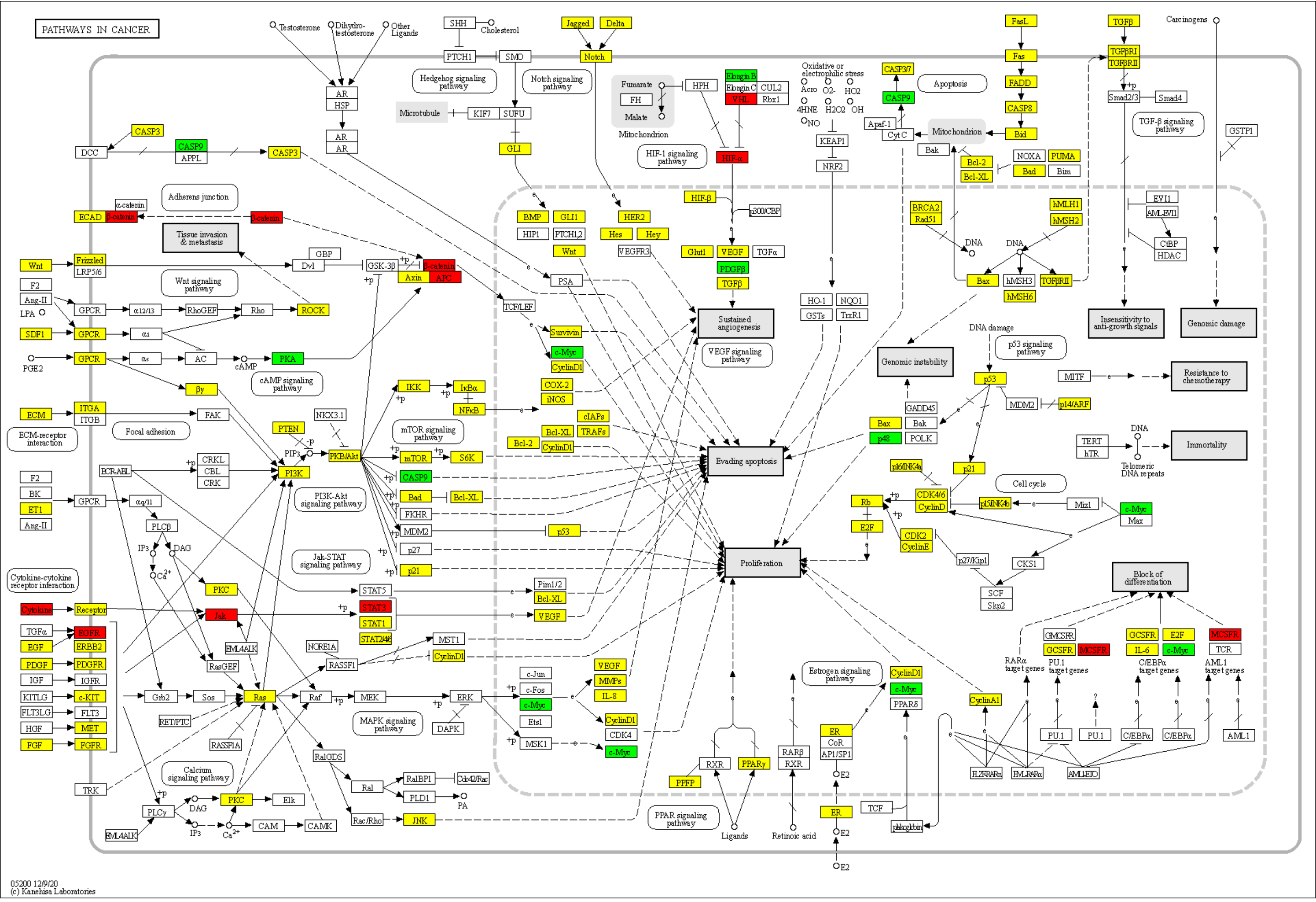

Supplementary Figure S3 B: Gene expression changes between EGFR-Ex20mut and EGFR/ERBB2wt tumors in the pathways in cancer system. Red = overexpression, green = underexpression, yellow = no significant change, white = human gene, but not covered by the targeted gene expression assay.

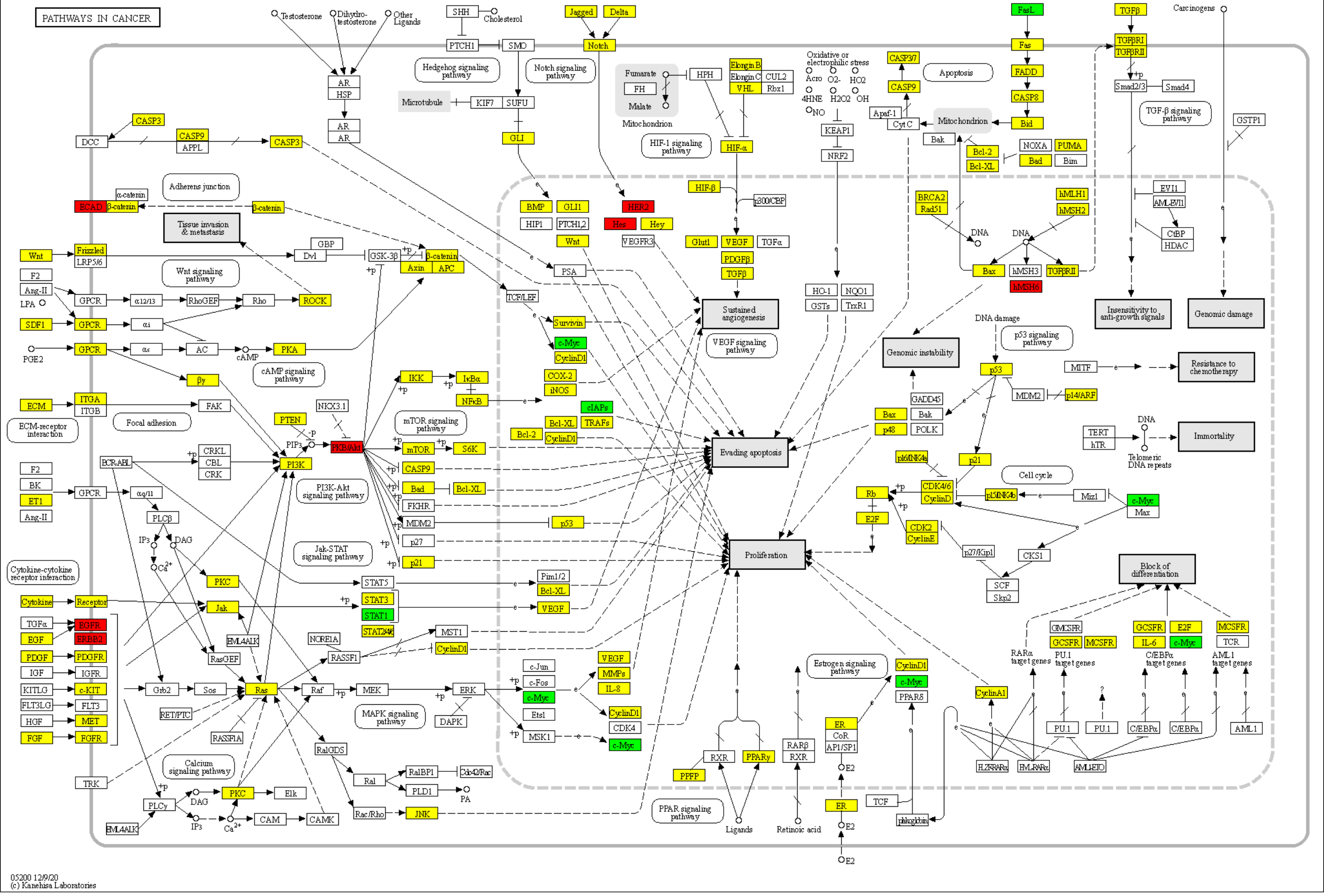

Supplementary Figure S3 C: Gene expression changes between EGFR-Ex18/19/21mut and EGFR/ERBB2wt tumors in the pathways in cancer system. Red = overexpression, green = underexpression, yellow = no significant change, white = human gene, but not covered by the targeted gene expression assay.

Supplementary Table S4 A: Gene expression changes between ERRB2-Ex20mut and EGFR/ERBB2wt tumors in the pathways in cancer system.

	Differentially expressed genes between ERRB2-Ex20mut and EGFR/ERBB2wt tumors		
Gene	***Name***	***Gene is assigned to pathway***	***Gene is***
PKA (PRKACA)	Protein Kinase CAMP-Activated Catalytic Subunit Alpha	Wnt signaling	downregulated
GPCR	G protein-coupled receptor	Wnt signaling	upregulated
CTNNB1 (ß-Catenin)	Catenin Beta 1	Wnt signaling	upregulated
AXIN	Axis inhibiting gene	Wnt signaling	upregulated
APC	Adenomatous Polyposis Coli	Wnt signaling	upregulated
PDGF	Platelet-derived growth factor	Cytokine-cytokine receptor interactions	downregulated
MET	MET Proto-Oncogene	Cytokine-cytokine receptor interactions	downregulated
ERBB2 (HER2)	Erb-b2 receptor tyrosine kinase 2	Cytokine-cytokine receptor interactions, NOTCH signaling	upregulated
cKIT	Proto-oncogene c-KIT	Cytokine-cytokine receptor interactions	upregulated
FASL	Fas Ligand	Apoptosis	downregulated
CASP8	Caspase 8	Apoptosis	downregulated
BRCA2	Breast Cancer Gene 2	Apoptosis	upregulated
PUMA (BBC3)	P53 upregulated modulator of apoptosis	Apoptosis	upregulated
BAD	BCL2 Associated Agonist Of Cell Death	mTOR signaling, Apoptosis	upregulated
S6K (RPS6KB1)	Ribosomal Protein S6 Kinase B1	mTOR signaling	upregulated
IKBA (NFKB1A)	NF-kappa-B inhibitor	mTOR signaling	upregulated
HIF-ß	Hypoxia-inducible factor 1 beta	HIF-1 signaling	upregulated
VHL	Von Hippel-Lindau Tumor Suppressor	HIF-1 signaling	upregulated
PDGFB	Platelet-derived growth factor beta	HIF-1 signaling	downregulated
AKT (AKT1)	AKT Serine/Threonine Kinase 1	PI3K-AKT signaling	upregulated
PI3K	Phosphoinositide 3-kinase	PI3K-AKT signaling	downregulated
JAK	Janus kinase	JAK-STAT signaling	upregulated
STAT3	Signal Transducer And Activator Of Transcription 3	JAK-STAT signaling	upregulated
CCNA1	Cyclin A1	Block of differentiation	downregulated
GCSFR (CD114)	Granulocyte colony-stimulating factor receptor	Block of differentiation	upregulated
HES	Hairy And Enhancer Of Split	NOTCH signaling	upregulated
ITGA	Integrin alpha	BCM-receptor interaction	downregulated
ET1 (EDN1)	Endothelin 1	BCM-receptor interaction	upregulated
cMYC (MYC)	MYC Proto-Oncogene	MAPK signaling, estrogen signaling, block of differentiation, cell cycle	downregulated
ECAD (CDH1)	Cadherin 1	Adherens junction	upregulated

Supplementary Tabele S4 B: Gene expression changes between EGFR-Ex20mut and EGFR/ERBB2wt tumors in the pathways in cancer system.

	Differentially expressed genes between EGFR-Ex20mut and EGFR/ERBB2wt tumors		
Gene	***Name***	***Gene is assigned to pathway***	***Gene is***
HIF-α	Hypoxia-inducible factor 1 beta	HIF-1 signaling	upregulated
VHL	Von Hippel-Lindau Tumor Suppressor	HIF-1 signaling	upregulated
PDGFB	Platelet-derived growth factor beta	HIF-1 signaling	downregulated
Elongin B (ELOB)	Elongin B	HIF-1 signaling	downregulated
PKA (PRKACA)	Protein Kinase CAMP-Activated Catalytic Subunit Alpha	Wnt signaling	downregulated
CTNNB1 (ß-Catenin)	Catenin Beta 1	Wnt signaling	upregulated
APC	Adenomatous Polyposis Coli	Wnt signaling	upregulated
JAK	Janus kinase	JAK-STAT signaling	upregulated
STAT3	Signal Transducer And Activator Of Transcription 3	JAK-STAT signaling	upregulated
CASP9	Caspase 9	Apoptosis	downregulated
cMYC (MYC)	MYC Proto-Oncogene	MAPK signaling, estrogen signaling, block of differentiation, cell cycle	downregulated
EGFR	Epidermal Growth Factor Receptor	Cytokine-cytokine receptor interactions	upregulated
MCSFR	Macrophage colony-stimulating factor receptor	Block of differentiation	upregulated
P48 (PTF1A)	Pancreas Associated Transcription Factor 1a	P53 signaling	downregulated

Supplementary Tabele S4 C: Gene expression changes between EGFR-Ex18/19/21mut and EGFR/ERBB2wt tumors in the pathways in cancer system.

	Differentially expressed genes between EGFR-Ex18/19/21mut and EGFR/ERBB2wt tumors		
Gene	***Name***	***Gene is assigned to pathway***	***Gene is***
FASL	Fas Ligand	Apoptosis	downregulated
hMSH6	MutS Homolog 6	Apoptosis	upregulated
cIAPs	Inhibitor of apoptosis proteins	Apoptosis	downregulated
EGFR	Epidermal Growth Factor Receptor	Cytokine-cytokine receptor interactions	upregulated
ERBB2 (HER2)	Erb-b2 receptor tyrosine kinase 2	Cytokine-cytokine receptor interactions, NOTCH signaling	upregulated
HES	Hairy And Enhancer Of Split	NOTCH signaling	upregulated
cMYC (MYC)	MYC Proto-Oncogene	MAPK signaling, estrogen signaling, block of differentiation, cell cycle	downregulated
STAT1	Signal Transducer And Activator Of Transcription 1	JAK-STAT signaling	downregulated
AKT (AKT1)	AKT Serine/Threonine Kinase 1	PI3K-AKT signaling	upregulated
ECAD (CDH1)	Cadherin 1	Adherens junction	upregulated

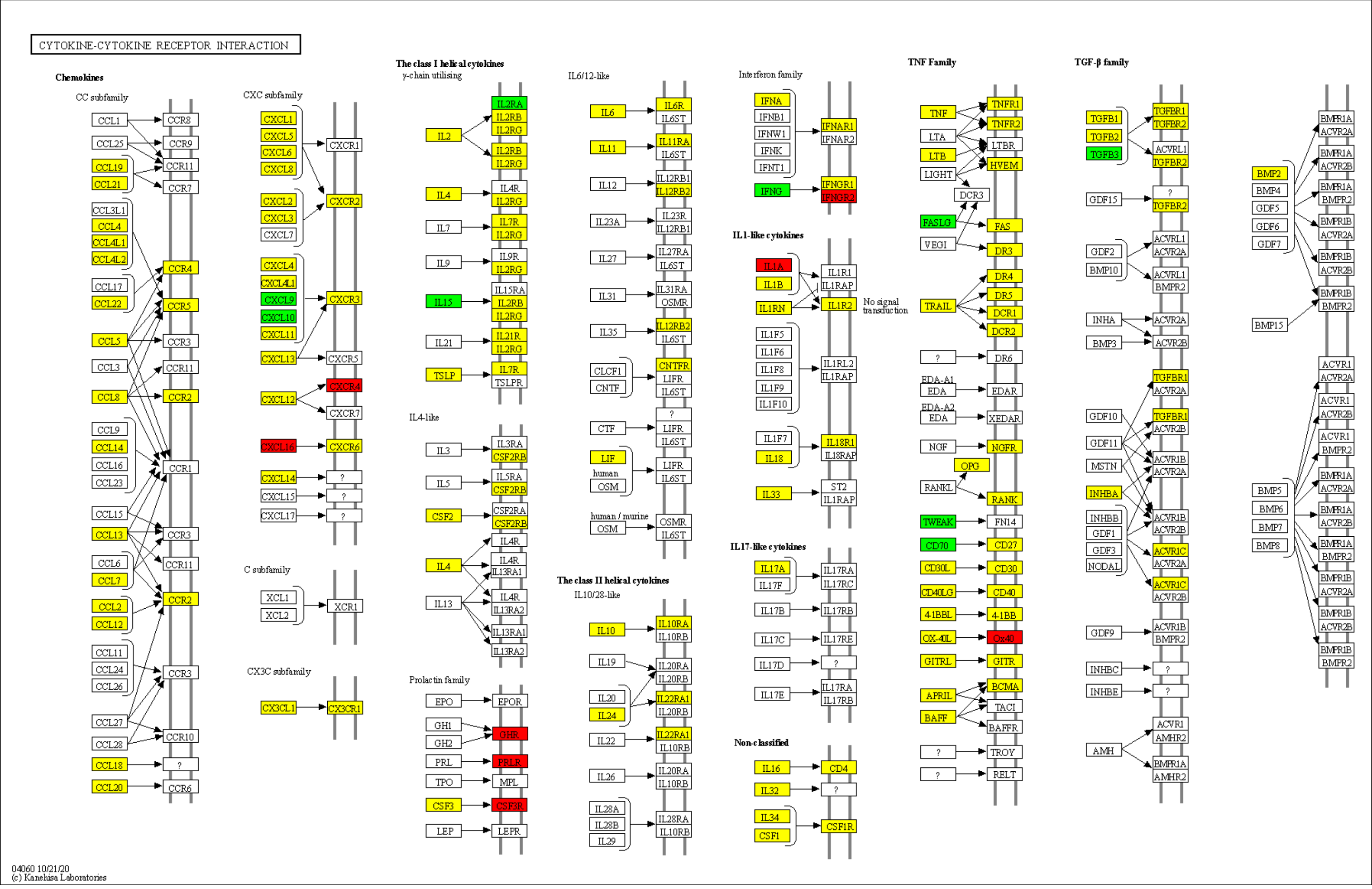

Supplementary Figure S4 A: Gene expression changes between ERBB2-Ex20 mut and EGFR/ERBB2wt tumors in the cytokine-cytokine receptor system. Red = overexpression, green = underexpression, yellow = no significant change, white = human gene, but not covered by the targeted gene expression assay.

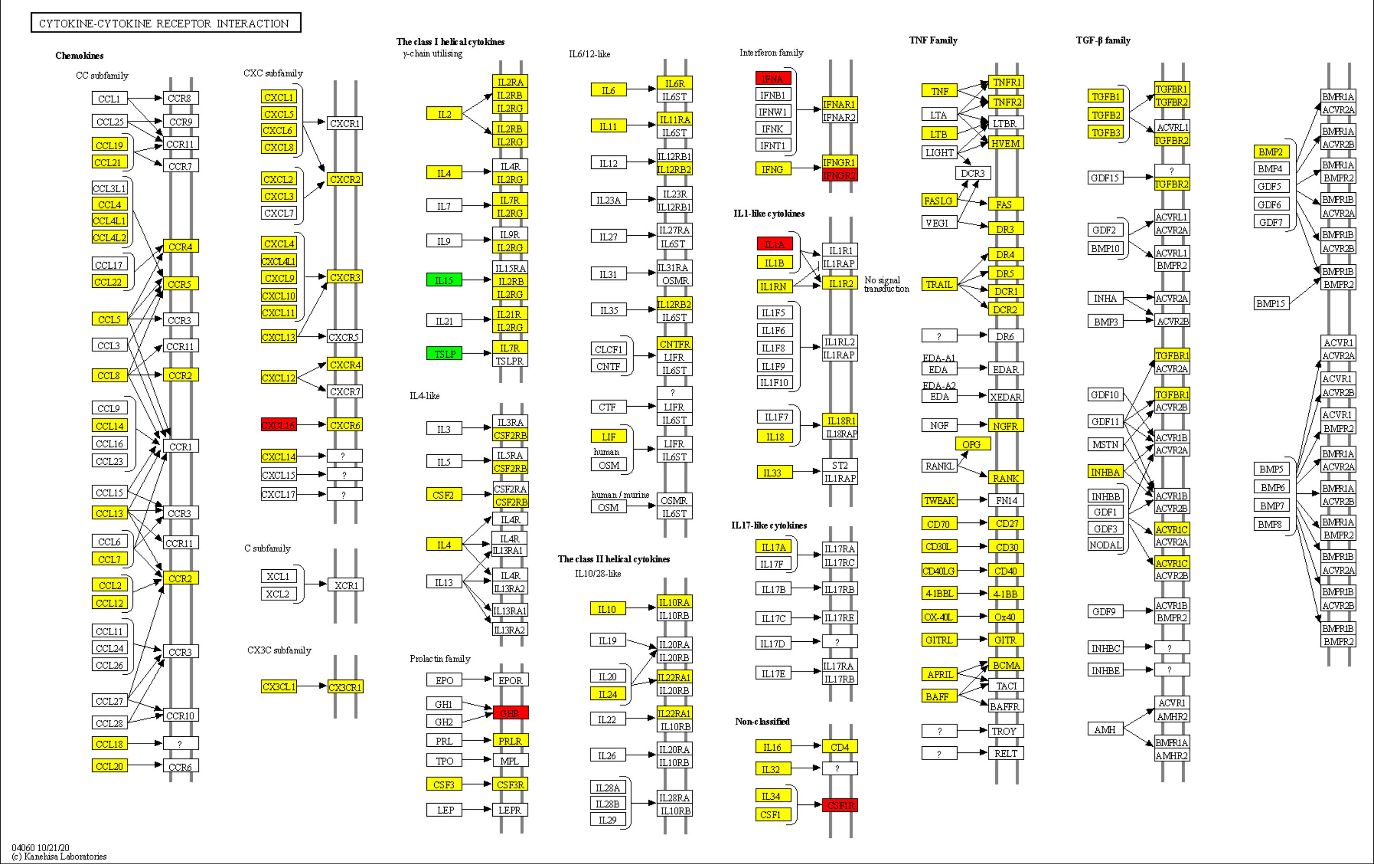

Supplementary Figure S4 B: Gene expression changes between EGFR-Ex20 mut and EGFR/ERBB2wt tumors in the cytokine-cytokine receptor system. Red = overexpression, green = underexpression, yellow = no significant change, white = human gene, but not covered by the targeted gene expression assay.

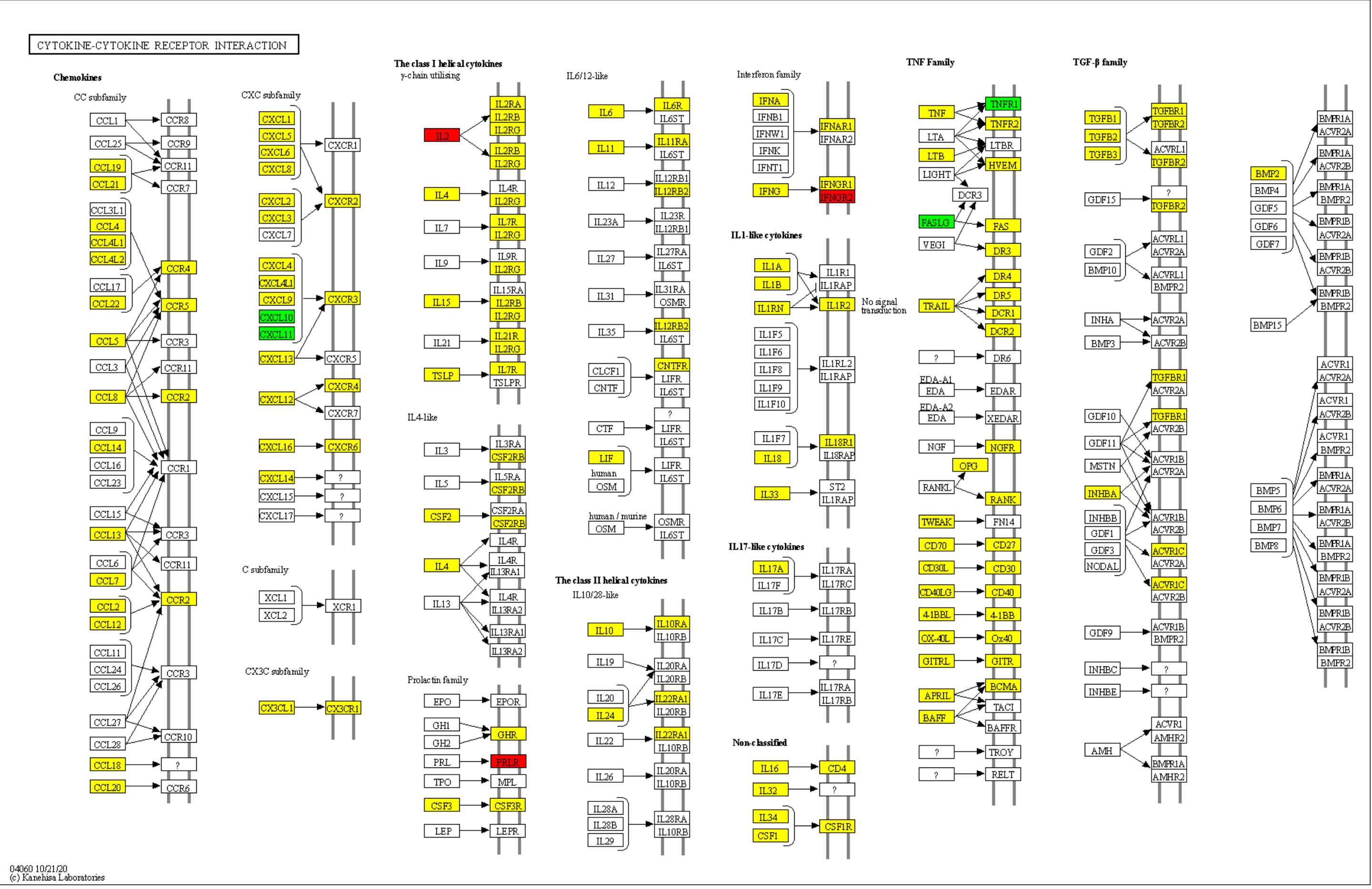

Supplementary Figure S4 C: Gene expression changes between EGFR-Ex18/19/21 mut and EGFR/ERBB2wt tumors in the cytokine-cytokine receptor system. Red = overexpression, green = underexpression, yellow = no significant change, white = human gene, but not covered by the targeted gene expression assay.
